# Interventional Effects and Neurometabolic Regulation of a *Panax quinquefolius*-Containing Herbal Extract (JYF) in Diazepam Withdrawal Syndrome in Mice

**DOI:** 10.3390/ph19101617

**Published:** 2026-10-12

**Authors:** Yue Jiao, Chang Gao, Peng Li, Yanjiao Liu, Xiaoliang Zhao, Yang Liu, Zhiguo Wang, Yinglan Nie, Tao Li

**Affiliations:** 1 Experimental Research Center, China Academy of Chinese Medical Sciences, Beijing 100700, China; 2 Yuyuantan Community Health Service Center of Haidian District, Beijing 100142, China; 3 Guang’anmen Hospital, China Academy of Chinese Medical Sciences, Beijing 100053, China

**Keywords:** diazepam withdrawal, *Panax quinquefolius*-containing herbal extract, network medicine, metabolomics, neurotransmitter metabolism, sleep architecture

## Abstract

**Background:** Long-term benzodiazepine use can lead to dependence and clinically significant withdrawal following discontinuation, highlighting the need to identify effective interventions for withdrawal-associated abnormalities. **Methods:** High-resolution mass spectrometry was used to characterize the constituents of a *Panax quinquefolius*-containing herbal extract (JYF) and evaluate their in vivo exposure in rats. Sixty-five C57BL/6 mice were randomly assigned to control, model, and low-, medium-, and high-dose JYF groups. Diazepam withdrawal was induced after 28 days of subcutaneous diazepam administration (12 mg/kg), and JYF was administered orally during the peri-withdrawal period for 7 days, beginning 4 days before diazepam discontinuation. Behavioral assessments, electroencephalographic/electromyographic (EEG/EMG) recordings, histopathological analysis, and neurochemical measurements were performed. Network proximity analysis, widely targeted metabolomics, targeted neurotransmitter quantification, and integrated pathway analysis were subsequently performed. **Results**: A total of 61 constituents were identified in JYF, of which 44 were detected in serum and 34 in brain tissue. Network proximity analysis highlighted genistein, columbamine, jatrorrhizine, and berberine as candidate constituents associated with withdrawal, anxiety/depression and sleep-related modules. JYF attenuated diazepam withdrawal-associated weight loss, anxiety-like behavior, sleep fragmentation, abnormal EEG activity, and elevations in corticosterone and orexin A. Metabolomic analyses revealed disturbances in lipid, energy, and amino acid metabolism, with tryptophan and phenylalanine metabolism emerging as prominent pathways associated with JYF intervention. Diazepam withdrawal also disrupted cerebral monoamine homeostasis, as reflected by increased serotonin and norepinephrine and decreased dopamine levels; these alterations generally shifted toward control levels after JYF intervention. **Conclusions**: JYF alleviates behavioral and sleep-related abnormalities associated with diazepam withdrawal. Its effects may be associated with alterations in brain metabolic homeostasis, including tryptophan- and phenylalanine-related metabolism and monoaminergic neurotransmission. Genistein and berberine were identified as candidate active constituents that warrant further experimental validation.

## 1. Introduction

BZD withdrawal is commonly characterized by rebound insomnia, pronounced anxiety, and, in severe cases, seizures. Neurological symptoms may persist for months in some patients. Although gradual dose tapering remains the mainstay of clinical management, it does not directly reverse the neuroadaptive changes induced by prolonged BZD exposure, and successful discontinuation remains challenging for some patients [1,2]. Therefore, there is a clear need for interventional strategies that can alleviate withdrawal symptoms and improve neurophysiological dysregulation.

The pathophysiology of benzodiazepine (BZD) withdrawal is complex and involves both large-scale neural network remodeling and disturbances in neurotransmitter systems. In a clinical study of long-term diazepam users, electroencephalographic (EEG) abnormalities were observed in 78.8% of patients who had taken diazepam for more than 6 months, compared with 57.6% in a control group without sedative use. The severity of these EEG abnormalities also increased with longer diazepam exposure [3]. BZDs exert their sedative and hypnotic effects primarily by enhancing γ-aminobutyric acid (GABA)-mediated inhibitory neurotransmission. However, chronic exposure induces compensatory reductions in GABAA receptor responsiveness and enhanced glutamatergic signaling involving receptors such as GluR1. Withdrawal may disrupt this adapted state, resulting in widespread excitation–inhibition imbalance [4]. Beyond classical GABA/glutamate dysregulation, neuroendocrine alterations involving the hypothalamic–pituitary–adrenal axis and orexin system, together with changes in synaptic plasticity, neuroinflammation, and mitochondrial function, may further contribute to withdrawal pathophysiology [5,6]. These interacting mechanisms collectively disrupt brain networks involved in reward and stress regulation, with neurotransmitter imbalance representing a key molecular basis underlying withdrawal-associated affective and somatic symptoms. Therefore, interventional strategies targeting neurotransmitter and neuroendocrine homeostasis represent an important direction for BZD withdrawal research [7].

Traditional Chinese medicine has a long history of use in the clinical management of drug dependence across East Asia, including China. The extensive clinical experience accumulated through their application provides a valuable source for identifying interventional candidates and developing complementary treatment strategies. *Panax quinquefolius*-containing herbal extract (JYF) was formulated and standardized by our research group. JYF originated from a traditional formula used by Lin Zexu (1785–1850) for opium withdrawal and was subsequently modified by Liu Yanjiao for the clinical management of insomnia associated with dependence on hypnotic medications. Standardized diagnostic and interventional protocols for JYF have since been established and adopted in clinical institutions in China [8]. Previous preclinical studies have suggested that JYF may ameliorate dependence-related abnormalities by modulating dopaminergic and glutamatergic neurotransmission and regulating mammalian target of rapamycin (mTOR) signaling activity [9]. These findings indicate that JYF may influence neuroadaptive processes associated with drug dependence. Moreover, pharmacological studies have demonstrated that major constituents derived from Gastrodia elata and American ginseng, including gastrodin, ginsenoside Rg1, and ginsenoside Rb1, exhibit diverse neuroactive properties, such as regulation of dopamine metabolism, improvement in sleep quality, and neuroprotective effects [10,11,12]. However, whether JYF can effectively alleviate diazepam withdrawal syndrome and associated manifestations, including rebound insomnia and anxiety-like symptoms, remains unclear. Furthermore, the neurochemical mechanisms and metabolic pathways underlying the effects of JYF on brain homeostasis require further investigation.

In this study, we established a mouse model of spontaneous withdrawal after prolonged diazepam exposure and systematically evaluated the effects of JYF on withdrawal-associated phenotypes. Behavioral testing and EEG/EMG recordings were combined with widely targeted brain metabolomics and targeted neurotransmitter quantification to characterize changes in sleep architecture, electrophysiological activity, and neurochemical and metabolic profiles. We further integrated in vivo chemical profiling with network medicine and pathway analysis to identify candidate constituents and biological processes associated with JYF intervention.

## 2. Results

### 2.1. Identification of the Major Chemical Constituents of JYF

The chemical constituents of the JYF extract and the absorbed constituents detected in rat serum and brain tissue after oral administration were characterized by HPLC–HRMS. Total ion chromatograms acquired in positive and negative ion modes are shown in Supplementary Figure S1. A total of 61 constituents were identified in the JYF aqueous extract, including 3 organic acids, 18 flavonoids, 18 saponins, 12 alkaloids, 2 terpenoid lactones, and 4 other constituents. Among these, 38 were confirmed by comparison with authentic reference standards. Of the 61 constituents identified in the extract, 44 were detected in serum and 34 in brain tissue (Table 1).

### 2.2. Compound–Target–Disease Network Pharmacology and Network Proximity Analysis of JYF

Among the 44 circulating JYF constituents, ChEMBL screening identified 13 constituents with experimentally supported targets that met the predefined inclusion criteria, yielding 71 unique constituent–target pairs. Of the resulting targets, 42 mapped to a high-confidence human physical protein–protein interaction (PPI) network and were retained for network proximity analysis.

**Figure 1:**
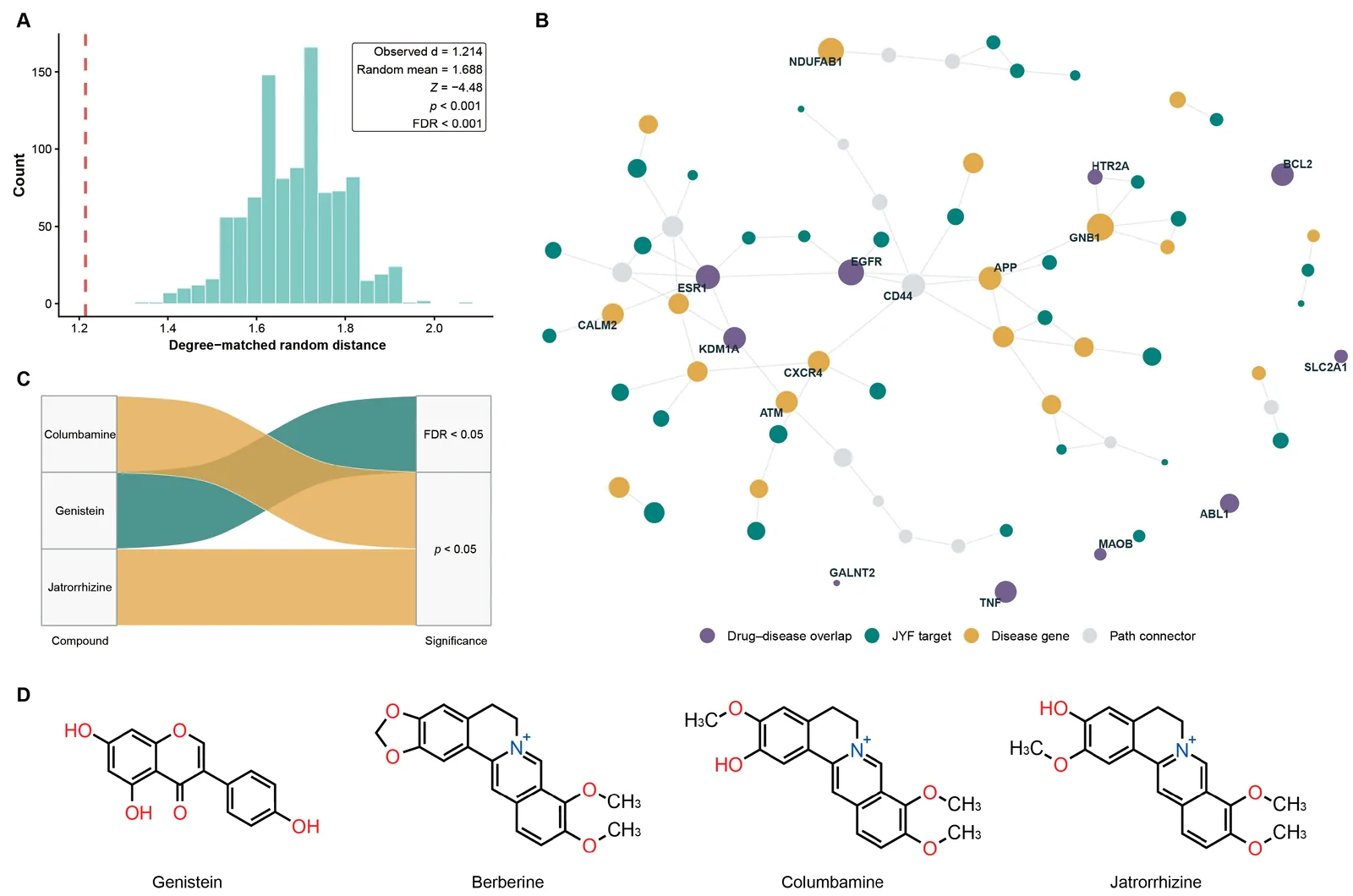
Network proximity analysis between targets of circulating JYF constituents and gene modules associated with diazepam withdrawal and related symptoms. (**A**) Degree- and set-size-matched permutation distributions of network distances between the pooled JYF target set and the corresponding disease modules. The red dashed line indicates the observed degree-matched random distance (observed d = 1.214); (**B**) Shortest-path subnetwork connecting JYF targets to their nearest disease-associated genes; (**C**) Network proximity between individual JYF constituents and the corresponding disease modules. Yellow flows indicate significance at *p* < 0.05, whereas green flows indicate significance at FDR < 0.05; (**D**) Two-dimensional chemical structures of the representative candidate constituents prioritized by network proximity analysis, including genistein, berberine, columbamine, and jatrorrhizine.

Using *p* < 0.05 as the significance threshold, the overall target set of circulating JYF constituents showed significant network proximity to all six disease/symptom modules, including the withdrawal-related (W) module, anxiety/depression-related (AD) module, sleep disorder-related (S) module, withdrawal–anxiety/depression shared (WAD) module, withdrawal–sleep disorder shared (WS) module, and withdrawal–anxiety/depression–sleep disorder (WADS) common module. Based on Z-scores, the modules showed the following degrees of network proximity: AD (Z = −4.881), WADS (Z = −4.475), WS (Z = −4.381), WAD (Z = −4.248), S (Z = −4.152), and W (Z = −3.921), with *p* < 0.001 for all comparisons. At the individual constituent level, genistein showed significant network proximity to all six modules (Z = −3.967 to −3.153, *p* < 0.01). Columbamine and jatrorrhizine showed significant proximity to the W, WAD, WS, and WADS modules (columbamine: Z = −3.752 to −3.458; jatrorrhizine: Z = −2.343 to −2.182; *p* < 0.05), whereas berberine showed significant proximity to the AD module (Z = −2.371, *p* < 0.05) (Figure 1 and Supplementary Figures S2–S6). These results indicate significant topological associations between JYF constituent targets and disease/symptom modules related to withdrawal, anxiety/depression, and sleep disturbances. Genistein, columbamine, jatrorrhizine, and berberine were therefore identified as candidate active constituents for further experimental investigation.

### 2.3. JYF Improves the General Condition of Mice with Diazepam Withdrawal

**Figure 2:**
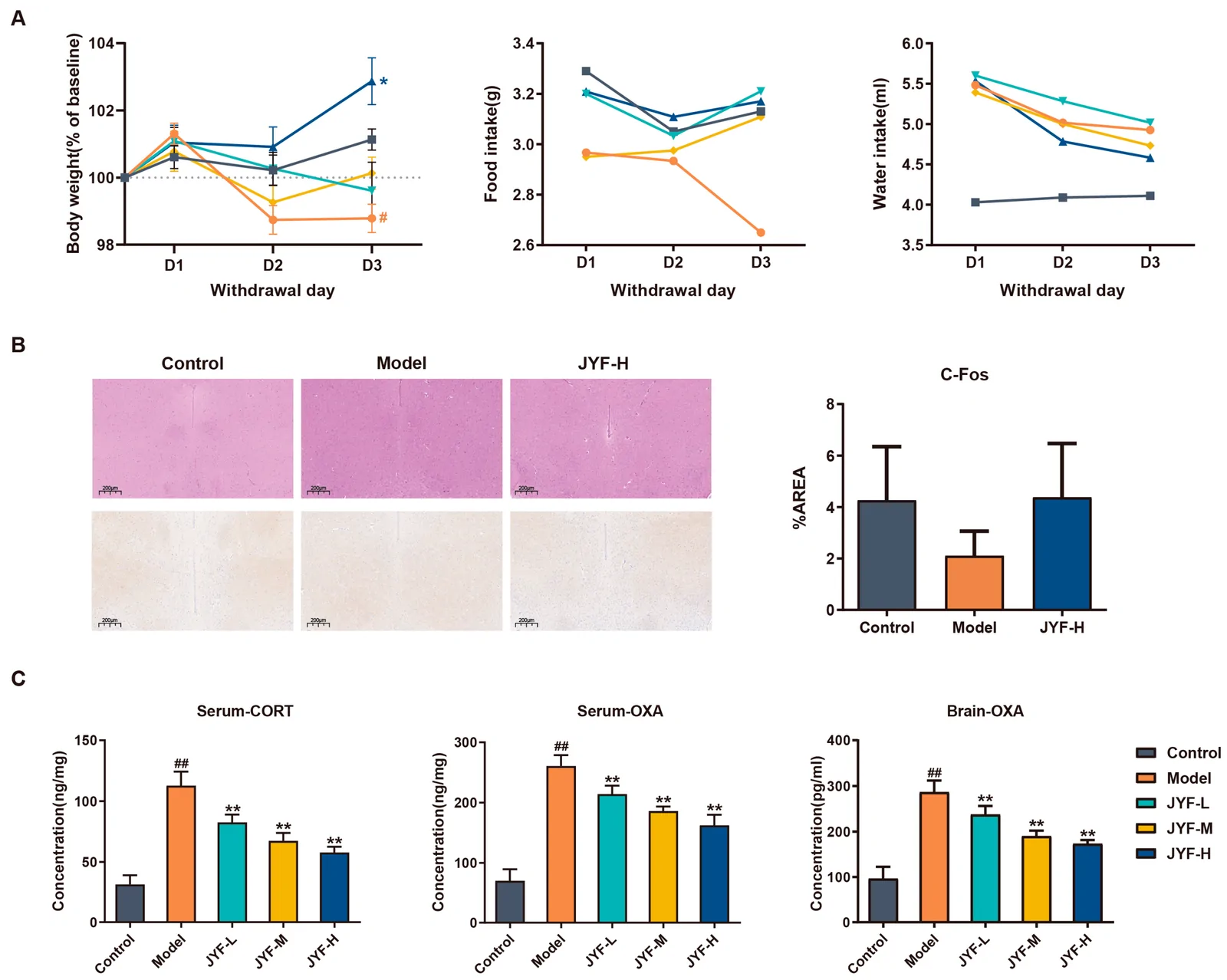
Effects of JYF on general physiological status, brain histopathology, and corticosterone and orexin A levels in mice undergoing diazepam withdrawal. (**A**) Changes in body weight, food intake, and water intake during diazepam withdrawal (*n* = 10); (**B**) hematoxylin and eosin (H&E) staining of midbrain tissue and quantitative analysis of c-Fos expression (*n* = 4); (**C**) serum CORT, serum OXA, and brain OXA levels (*n* = 10). Compared with the normal control group, ^#^ *p* < 0.05, ^##^ *p* < 0.01; compared with the model group, * *p* < 0.05, ** *p* < 0.01.

Body weight did not differ significantly among groups before diazepam withdrawal. After withdrawal, body weight progressively declined in the model group and reached 98.57% of baseline on day 3, a value significantly lower than that in the control group (*p* < 0.05). JYF attenuated this weight loss. The effect was most pronounced in the high-dose group, in which body weight reached 102.57% of baseline on day 3 and was significantly higher than that in the model group (*p* < 0.05). The model group also showed reduced food intake and increased water intake during withdrawal (*p* < 0.05). Both changes tended to improve after JYF treatment, although the differences from the model group were not statistically significant (Figure 2A).

### 2.4. JYF Aqueous Extract Improves Locomotor Function and Anxiety-like Behaviors in Mice with Diazepam Withdrawal

**Figure 3:**
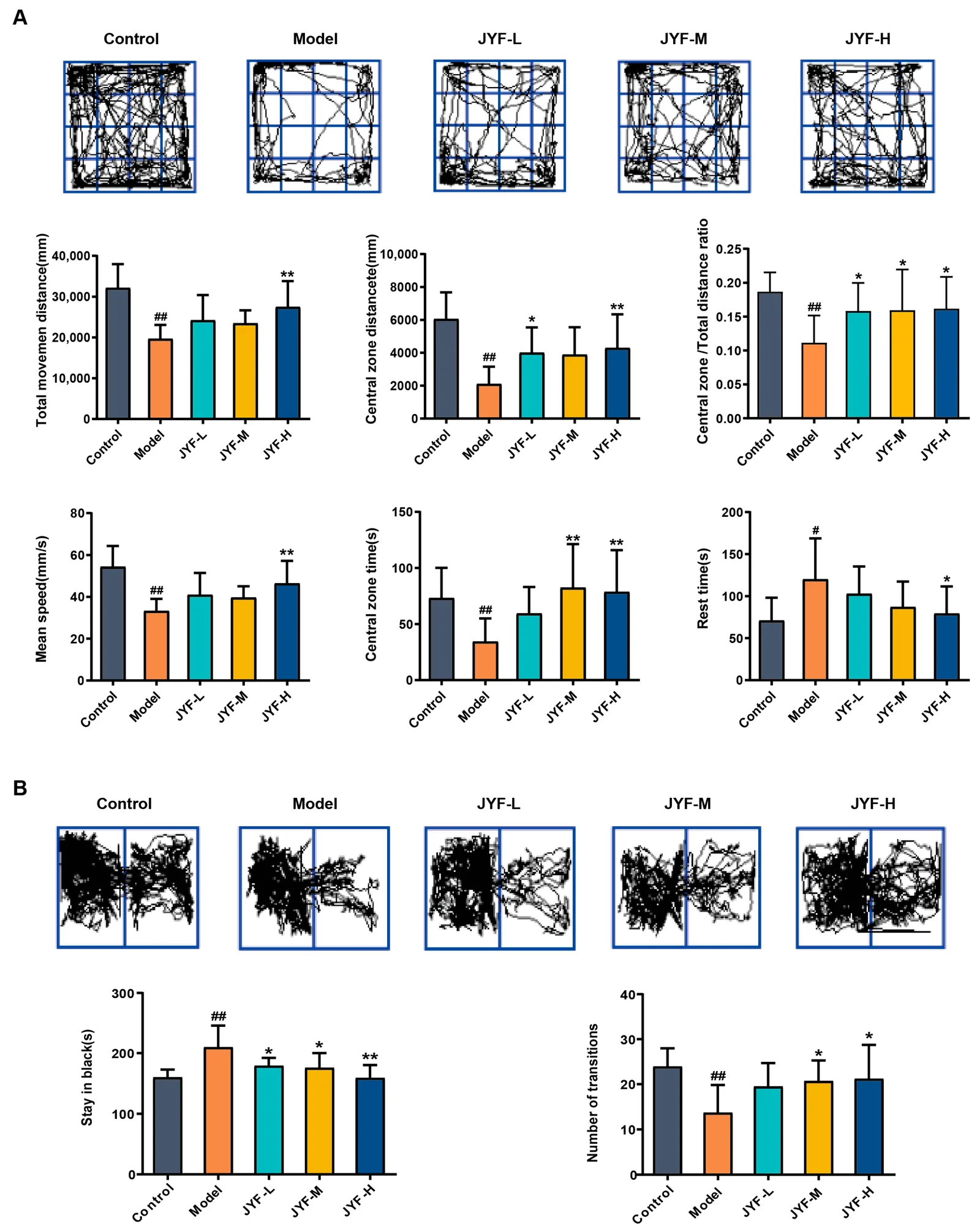
Effects of JYF on locomotor activity and anxiety-like behaviors in mice undergoing diazepam withdrawal (*n* = 10). (**A**) Representative movement trajectories and behavioral parameters from the open-field test, including total movement distance, central zone distance, central zone distance/total distance ratio, mean speed, central zone time, and rest time; (**B**) Representative movement trajectories and behavioral parameters from the light–dark box test, including time spent in the dark compartment and number of transitions. Compared with the normal control group, ^#^ *p* < 0.05, ^##^ *p* < 0.01; compared with the model group, * *p* < 0.05, ** *p* < 0.01.

In the open-field test, mice in the model group showed significant reductions in total movement distance, central zone distance, the central zone distance/total distance ratio, mean speed, and central zone time compared with control mice (*p* < 0.01), together with an increase in rest time (*p* < 0.05) (Figure 3A). These changes indicate reduced spontaneous locomotor and exploratory activity, together with reduced relative exploration of the central zone during diazepam withdrawal. Compared with the model group, the JYF-H group showed significant increases in total movement distance, central zone distance, mean speed, and central zone time (*p* < 0.01), along with an increased central zone distance/total distance ratio (*p* < 0.05) and reduced rest time (*p* < 0.05). Central zone distance was also increased in the JYF-L group (*p* < 0.05), while the central zone distance/total distance ratio was increased in both the JYF-L and JYF-M groups (*p* < 0.05). Central zone time was also increased in the JYF-M group (*p* < 0.01). Overall, JYF intervention attenuated several withdrawal-associated changes in locomotor and exploratory behavior. The increased central zone distance/total distance ratio also suggests greater relative central zone exploration after JYF intervention, although open-field measures should be interpreted in the context of overall locomotor activity.

In the light–dark box test, mice in the model group spent significantly more time in the dark compartment and made fewer transitions than control mice (*p* < 0.01), consistent with increased anxiety-like behavior during diazepam withdrawal (Figure 3B). JYF significantly reduced the time spent in the dark compartment at all three doses (*p* < 0.05 for JYF-L and JYF-M; *p* < 0.01 for JYF-H). The number of transitions was also significantly increased in the JYF-M and JYF-H groups (*p* < 0.05). Taken together, the open-field and light–dark box findings suggest that JYF attenuated withdrawal-associated anxiety-like behavioral changes while also attenuating several withdrawal-associated reductions in spontaneous locomotor and exploratory activity.

### 2.5. JYF Ameliorates Brain Functional Abnormalities in Mice Undergoing Diazepam Withdrawal

**Figure 4:**
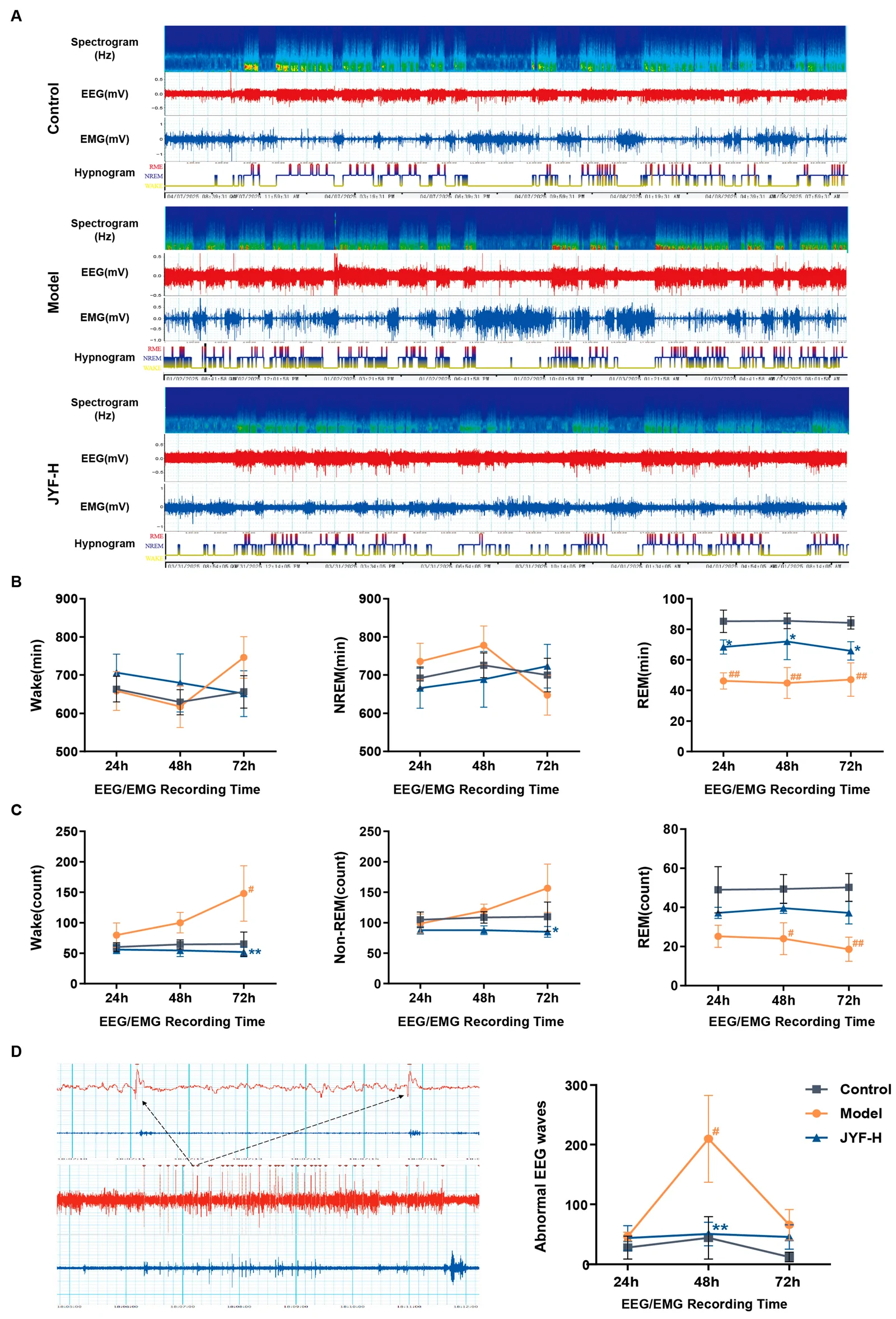
Effects of JYF on sleep architecture and abnormal EEG activity in mice undergoing diazepam withdrawal (*n* = 5). (**A**) Representative sleep recordings from the control, model, and JYF-H groups, including the EEG spectrogram; (**B**) Durations of wake, non-rapid eye movement (NREM) sleep, and rapid eye movement (REM) sleep at 24, 48, and 72 h; (**C**) Numbers of wake, NREM, and REM episodes at 24, 48, and 72 h; (**D**) Representative abnormal EEG activity during diazepam withdrawal and quantitative analysis of the number of abnormal EEG events at 24, 48, and 72 h. Black dashed arrows indicate the magnified views of representative abnormal EEG events from the original recordings. Compared with the normal control group, ^#^ *p* < 0.05, ^##^ *p* < 0.01; compared with the model group, * *p* < 0.05, ** *p* < 0.01.

Sleep–wake monitoring revealed marked alterations in sleep regulation following diazepam withdrawal. Compared with the normal control group, mice in the model group showed a progressive reduction in total sleep time at 24, 48, and 72 h after withdrawal, accompanied by a corresponding increase in wake time. Analysis of sleep architecture showed that non-rapid eye movement (NREM) sleep proportion transiently increased at 48 h but subsequently declined, whereas rapid eye movement (REM) sleep was significantly reduced at 72 h. In addition, both wake and sleep episodes increased progressively, with pronounced fragmentation of NREM sleep characterized by shorter episode duration and increased episode frequency, indicating impaired sleep continuity during withdrawal. Compared with the model group, JYF treatment significantly increased total sleep time and partially restored sleep–wake organization, particularly by increasing REM sleep duration (*p* < 0.01). JYF also reduced the number of wake and sleep episodes, with the most prominent reduction observed in NREM sleep episodes (*p* < 0.01, *p* < 0.05) (Figure 4A–C). EEG analysis further showed that, compared with the normal control group, withdrawal mice exhibited abnormal spike-wave discharges resembling absence seizure-like activity, which occurred predominantly during sleep and were occasionally accompanied by mild myoclonic movements. The frequency of these abnormal EEG events significantly increased at 48 h after withdrawal (*p* < 0.05) and subsequently declined at 72 h. JYF treatment markedly reduced the occurrence of abnormal EEG discharges (*p* < 0.01) (Figure 4D). Together, these findings demonstrate that JYF alleviates diazepam withdrawal-associated disturbances in sleep architecture, reduces sleep fragmentation, and suppresses abnormal EEG activity.

### 2.6. Effects of JYF on Brain Histomorphology and Neuronal Activity

H&E staining showed preserved midbrain architecture in the control, model, and JYF-treated groups. Neuronal morphology remained regular, cytoplasmic and nuclear boundaries were clearly distinguishable, and no obvious edema or vascular congestion was observed. No overt histomorphological differences were detected among the groups (Figure 2B).

Immunohistochemical analysis showed a nonsignificant decrease in c-Fos expression in the model group relative to the control group and a nonsignificant increase after JYF treatment. Overall, c-Fos expression did not differ significantly among groups (Figure 2B).

### 2.7. JYF Attenuates Elevations in Serum Corticosterone and Orexin a and Brain Orexin a During Diazepam Withdrawal

Biochemical analysis showed that serum CORT, serum OXA, and brain OXA levels were significantly increased in the model group compared with the control group (*p* < 0.01). Compared with the model group, all three doses of JYF significantly reduced these parameters (*p* < 0.01), with an overall further decrease as the JYF dose increased, indicating an apparent dose–response relationship (Figure 2C).

### 2.8. JYF Regulates Brain Metabolic Disturbances and Neurotransmitter Imbalance in Mice with Diazepam Withdrawal

#### 2.8.1. Widely Targeted Metabolomic Analysis

**Figure 5:**
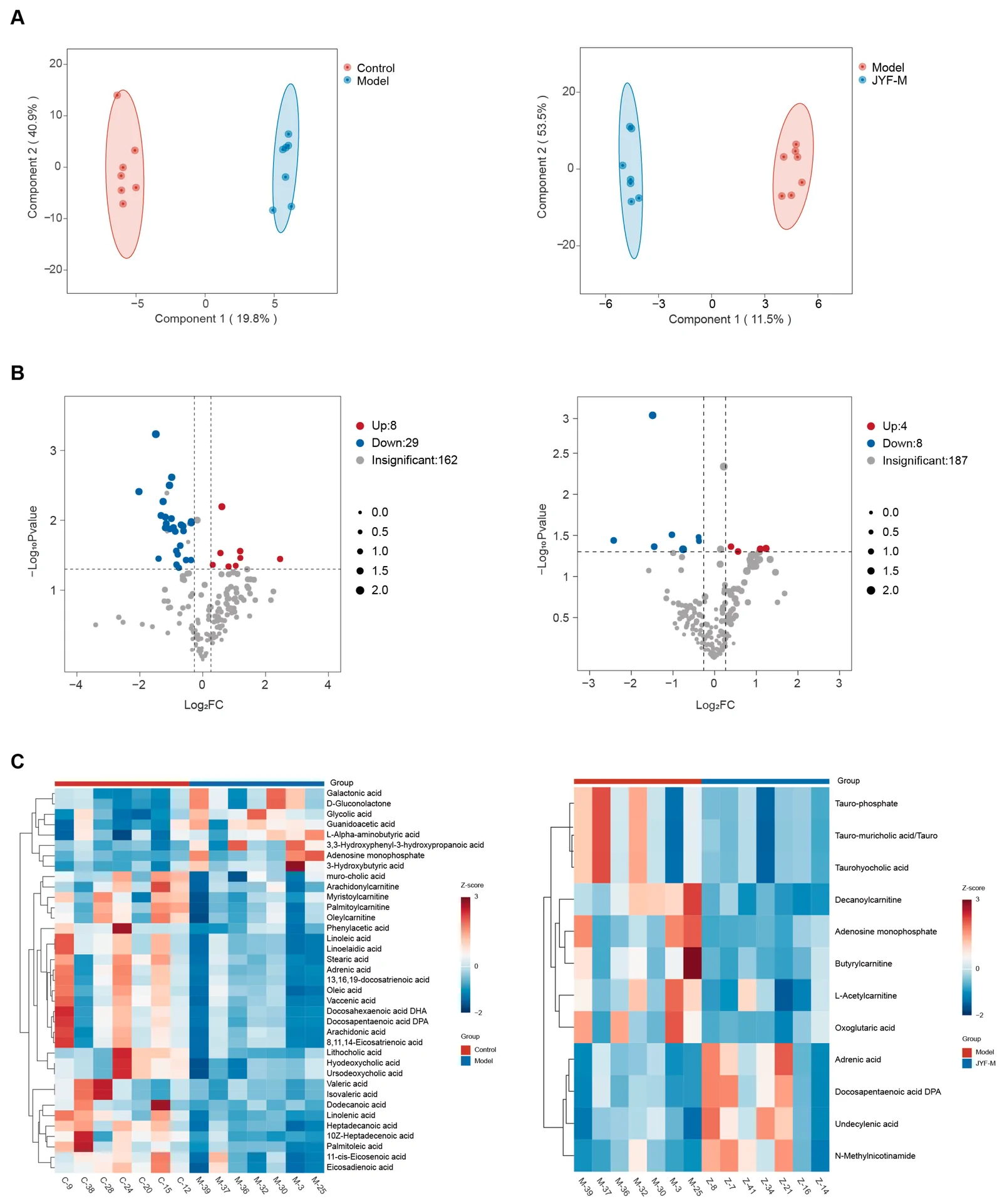
Widely targeted metabolomic profiling of brain tissue in mice undergoing diazepam withdrawal and JYF intervention. (**A**) OPLS-DA score plots of brain metabolite profiles for the control versus model comparison and the JYF-M versus model comparison; (**B**) Volcano plots of differential metabolites for the control versus model comparison and the JYF-M versus model comparison. Vertical and horizontal dashed lines indicate the thresholds for fold change (FC > 1.20 or FC < 0.83) and significance (*p* < 0.05), respectively. Red dots represent up-regulated metabolites, blue dots represent down-regulated metabolites, and gray dots represent non-significant metabolites; (**C**) Hierarchical clustering heatmaps of differential metabolites identified in the two comparisons.

Widely targeted metabolomic analysis was performed on brain tissues from each group. The principal component analysis (PCA) results for all groups are shown in Supplementary Figure S7. The PCA score plot showed tight clustering of the quality control (QC) samples, indicating good stability of the analytical system and high quality and reliability of the metabolomic data. Orthogonal partial least squares-discriminant analysis (OPLS-DA) was performed for the model versus control comparison and the JYF versus model comparison. Clear separation was observed between the model and control groups and between the JYF and model groups (Figure 5A).

Using the criteria VIP > 1, *p* < 0.05, and fold change (FC) > 1.20 or FC < 0.83, 37 differential metabolites were identified between the model and control groups, including 8 increased and 29 decreased metabolites. These metabolites mainly comprised fatty acids and their derivatives, acylcarnitines, bile acids, organic acids, and nucleotides. Representative changes included adenosine monophosphate (AMP), arachidonylcarnitine, oleylcarnitine, palmitoylcarnitine, myristoylcarnitine, arachidonic acid, linoleic acid, α-linolenic acid, adrenic acid, docosahexaenoic acid (DHA), docosapentaenoic acid (DPA), and several bile acids. Twelve differential metabolites were identified between the JYF and model groups, including 4 increased and 8 decreased metabolites. These included AMP, butyrylcarnitine, decanoylcarnitine, L-acetylcarnitine, N-methylnicotinamide, oxoglutaric acid, adrenic acid, DPA, and taurine-conjugated bile acids (Figure 5B,C). AMP, adrenic acid, and DPA were differential in both comparisons, suggesting that they may be associated with withdrawal-related metabolic disturbances and the response to JYF. Detailed results are provided in Supplementary Figure S1.

**Figure 6:**
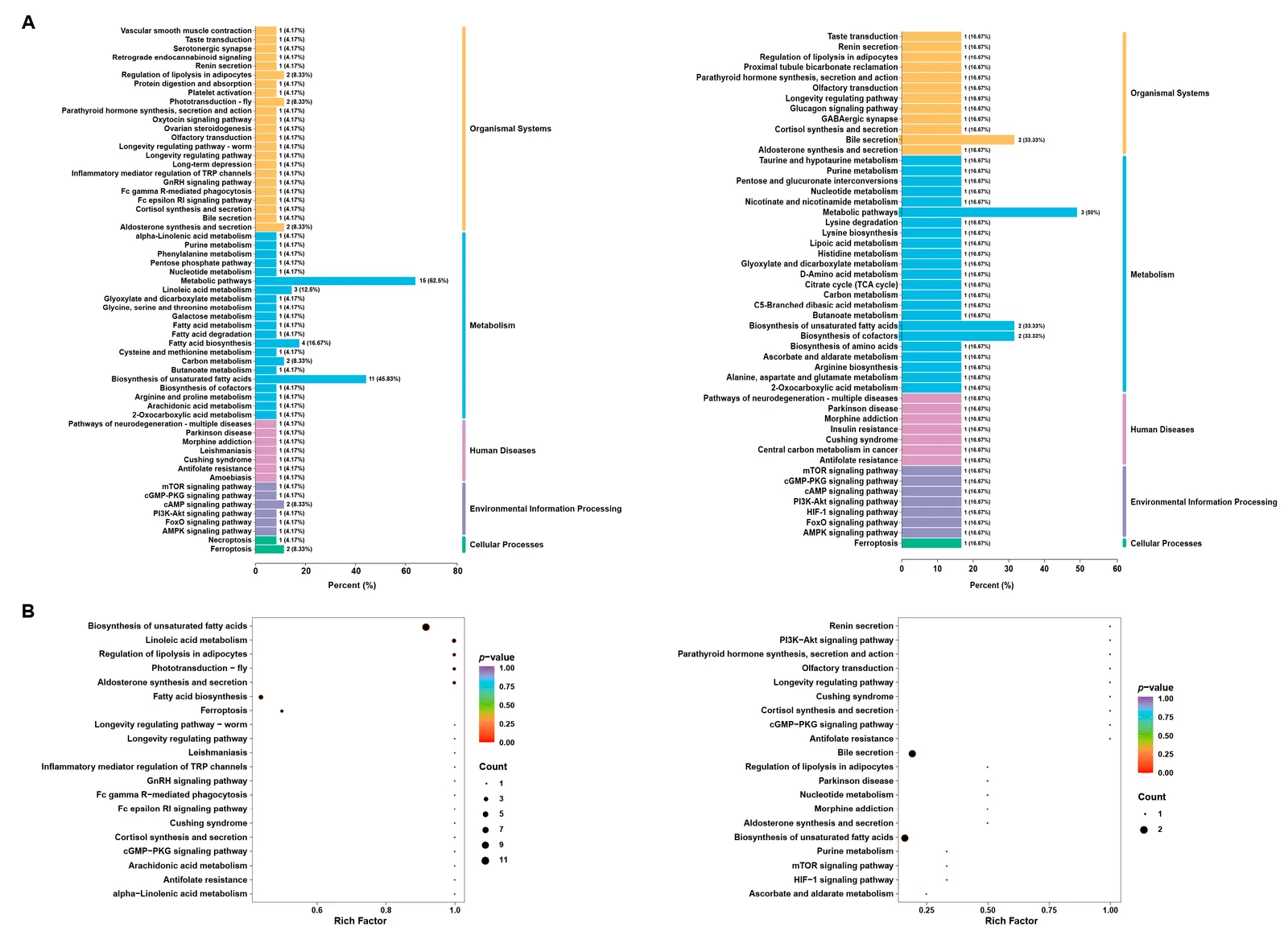
Functional classification and pathway enrichment analysis of differential brain metabolites. (**A**) KEGG functional classification of differential metabolites identified in the control versus model comparison and the JYF-M versus model comparison; (**B**) KEGG pathway enrichment analysis of differential metabolites identified in the two comparisons.

KEGG analysis indicated that metabolites altered by diazepam withdrawal were mainly associated with carbon metabolism, 2-oxocarboxylic acid metabolism, fatty acid metabolism and biosynthesis, arachidonic acid metabolism, linoleic acid metabolism, α-linolenic acid metabolism, biosynthesis of unsaturated fatty acids, purine metabolism, and several amino acid pathways (Figure 6A,B). These changes suggest broad disturbances in brain energy, lipid, nucleotide, and amino acid metabolism. Metabolites altered by JYF treatment were enriched in carbon metabolism, the tricarboxylic acid (TCA) cycle, 2-oxocarboxylic acid metabolism, butanoate metabolism, purine metabolism, amino acid biosynthesis and metabolism, nicotinate and nicotinamide metabolism, taurine and hypotaurine metabolism, biosynthesis of unsaturated fatty acids, and lipoic acid metabolism. Carbon metabolism, 2-oxocarboxylic acid metabolism, purine metabolism, and biosynthesis of unsaturated fatty acids were represented in both comparisons. Collectively, these findings suggest that JYF modulates withdrawal-associated disturbances in energy, lipid, amino acid, nucleotide, and cofactor metabolism.

#### 2.8.2. Targeted Quantification of Neurotransmitters and Related Metabolites

**Figure 7:**
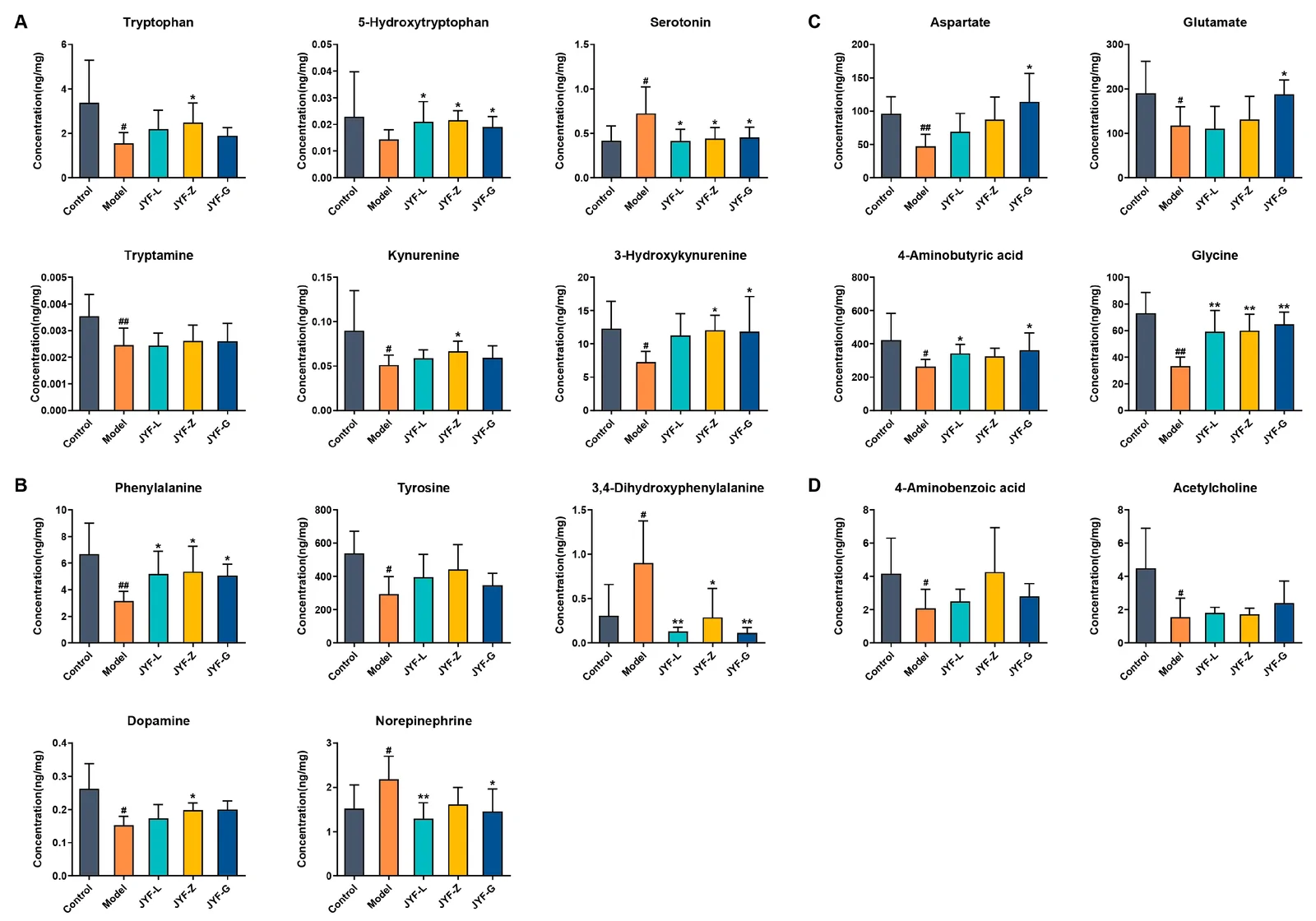
Effects of JYF on brain neurotransmitters and related metabolites in mice undergoing diazepam withdrawal. (**A**) Tryptophan–serotonin/melatonin metabolic pathway; (**B**) Phenylalanine–tyrosine–dopamine–norepinephrine metabolic pathway; (**C**) Amino acid neurotransmitters; (**D**) Other neurotransmitters and related metabolites. Compared with the normal control group, ^#^ *p* < 0.05, ^##^ *p* < 0.01; compared with the model group, * *p* < 0.05, ** *p* < 0.01.

Based on the results of widely targeted metabolomics, more than 40 neurotransmitters and related metabolites were further quantified using targeted analysis, with a focus on key neurotransmitter-associated pathways, including tryptophan–serotonin/melatonin metabolism, phenylalanine–tyrosine–dopamine–norepinephrine metabolism, and glutamine–glutamate–GABA metabolism (Figure 7). Compared with the control group, mice subjected to diazepam withdrawal exhibited significantly decreased levels of tryptophan, tryptamine, kynurenine, 3-hydroxykynurenine, aspartate, glutamate, GABA, glycine, phenylalanine, tyrosine, dopamine, 4-aminobenzoic acid, and acetylcholine in the brain, whereas serotonin, 3,4-dihydroxyphenylalanine (L-DOPA), and norepinephrine levels were significantly increased. These alterations indicated substantial disturbances in tryptophan metabolism, catecholamine metabolism, and excitatory/inhibitory amino acid neurotransmitter homeostasis. Following JYF treatment, the levels of tryptophan, 5-hydroxytryptophan, serotonin, kynurenine, 3-hydroxykynurenine, phenylalanine, L-DOPA, dopamine, norepinephrine, aspartate, glutamate, GABA, and glycine were altered to varying degrees, with several metabolites showing significant shifts toward control levels. These findings suggest that JYF may alleviate diazepam withdrawal-associated neurotransmitter dysregulation by modulating tryptophan metabolism, catecholamine metabolism, and excitatory/inhibitory amino acid neurotransmitter homeostasis.

### 2.9. Integrated Pathway and Correlation Analyses of JYF Targets, Differential Metabolites, and Neurotransmitters

**Figure 8:**
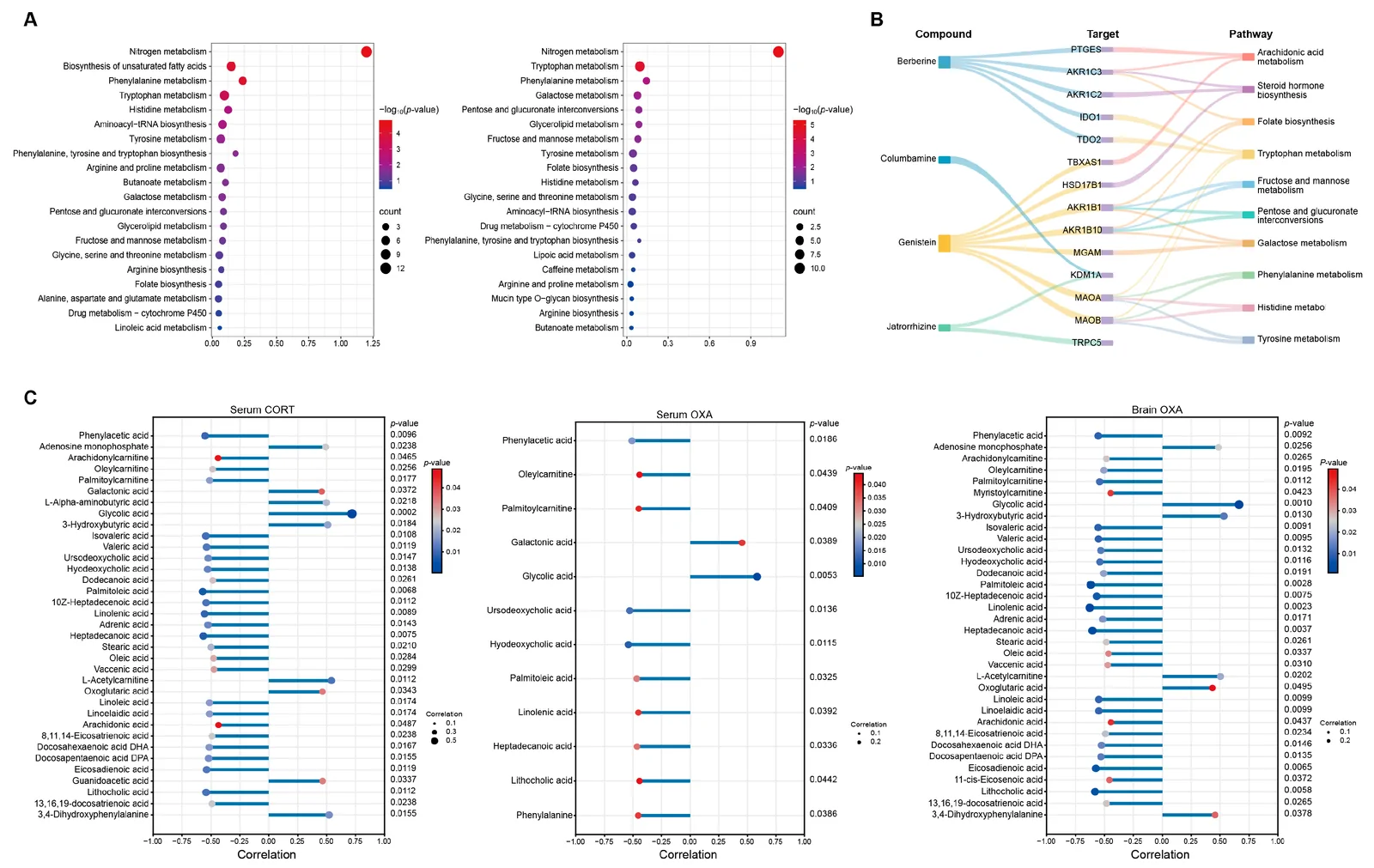
Integrated pathway analysis of JYF targets, differential metabolites, and neurotransmitters, and correlation analysis with CORT/OXA levels. (**A**) Joint pathway enrichment analysis integrating JYF candidate targets with differential metabolites and neurotransmitters identified in the model versus control and JYF-treated versus model comparisons; (**B**) JYF candidate constituent–target–metabolic pathway network; (**C**) Correlation analysis between differential metabolites and serum CORT, serum OXA, and brain OXA levels.

Using the Joint Pathway Analysis module of MetaboAnalyst 6.0, 57 ChEMBL-screened and standardized candidate JYF targets were integrated with differential metabolites and neurotransmitters from the control versus model and JYF versus model comparisons. The control versus model comparison was mainly enriched in nitrogen metabolism, biosynthesis of unsaturated fatty acids, phenylalanine metabolism, tryptophan metabolism, and histidine metabolism. The JYF versus model comparison was mainly enriched in nitrogen metabolism, tryptophan metabolism, phenylalanine metabolism, aminoacyl-tRNA biosynthesis, and histidine metabolism (Figure 8A). Nitrogen, tryptophan, phenylalanine, and histidine metabolism were enriched in both comparisons, suggesting that these pathways are shared between withdrawal-associated metabolic disturbances and metabolic changes associated with JYF intervention.

A JYF constituent–target–metabolic pathway network was further constructed based on the candidate constituents prioritized by network proximity analysis, including berberine, genistein, columbamine, and jatrorrhizine. The integrated network showed that berberine was connected with tryptophan metabolism through the candidate targets *IDO1* and *TDO2*, whereas genistein was associated with tryptophan, phenylalanine, histidine, and tyrosine metabolism through *MAOA* and *MAOB*. In addition, candidate constituents and their predicted targets were connected with arachidonic acid metabolism, steroid hormone biosynthesis, and several carbohydrate metabolism-related pathways (Figure 8B). The candidate targets of columbamine and jatrorrhizine were not directly mapped to the enriched metabolic pathways identified in this analysis; therefore, no corresponding target–pathway connections were established within this network.

Correlation analysis was further performed to evaluate the relationships between metabolomics-derived differential metabolites and CORT/OXA (Figure 8C). Notably, all three commonly reversed metabolites identified in the preceding metabolomic analysis were significantly correlated with CORT/OXA-related measures. AMP was positively correlated with serum CORT (ρ = 0.491, *p* = 0.024) and brain OXA (ρ = 0.486, *p* = 0.026), whereas adrenic acid was negatively correlated with serum CORT (ρ = −0.526, *p* = 0.014) and brain OXA (ρ = −0.514, *p* = 0.017); DPA was also negatively correlated with serum CORT (ρ = −0.521, *p* = 0.015) and brain OXA (ρ = −0.530, *p* = 0.013). In addition, glycolic acid showed strong positive correlations with serum CORT (ρ = 0.719, *p* < 0.001) and brain OXA (ρ = 0.664, *p* = 0.001), whereas brain OXA showed strong negative correlations with linolenic acid (ρ = −0.627, *p* = 0.002), palmitoleic acid (ρ = −0.618, *p* = 0.003), and heptadecanoic acid-1 (ρ = −0.604, *p* = 0.004). These results indicate that AMP, adrenic acid, and DPA, which were shifted toward control levels after JYF intervention, were strongly associated with changes in CORT/OXA, supporting their consideration as candidate pharmacodynamic metabolic markers associated with the response to JYF.

## 3. Discussion

In the present study, diazepam discontinuation produced sustained weight loss, reduced food intake, increased water intake, anxiety-like behavior, reduced locomotor and exploratory activity, sleep fragmentation, abnormal spike-wave EEG discharges, and elevated serum CORT and serum and brain OXA levels. Together, these findings support the establishment of a spontaneous diazepam withdrawal model characterized by behavioral, electrophysiological, and neuroendocrine abnormalities relevant to BZD withdrawal. JYF attenuated withdrawal-associated weight loss and anxiety-like behavior, improved sleep continuity, reduced abnormal EEG activity, and lowered CORT and OXA levels. Despite these functional alterations, H&E staining revealed no overt histomorphological abnormalities in the midbrain, and c-Fos expression did not differ significantly among groups. Previous studies have reported altered functional brain connectivity in individuals with methamphetamine or opioid use [13] and extensive reorganization of brain functional networks during psychostimulant withdrawal [14]. These observations suggest that early withdrawal may involve functional network and neurochemical alterations before overt structural injury becomes detectable. We therefore further examined brain metabolic and neurochemical changes to characterize the biological processes associated with diazepam withdrawal and the response to JYF.

High-resolution mass spectrometry combined with target screening identified 44 candidate constituents of JYF, 34 of which were detected in both serum and brain tissue. High-confidence candidate targets, including *HTR2A*, *MAOB*, *TNF*, and *EGFR*, were associated with processes related to emotional regulation, cognitive function, inflammatory responses, and cellular proliferation. Network proximity analysis showed significant topological proximity between the overall target set of circulating JYF constituents and disease modules related to withdrawal syndrome, anxiety/depression-related conditions, and sleep disorders (Z = −4.881 to −3.921). At the individual constituent level, genistein showed significant proximity to all six disease modules. Columbamine and jatrorrhizine showed significant proximity to the withdrawal syndrome module, the withdrawal–anxiety/depression shared module, the withdrawal–sleep disorder shared module, and the withdrawal–anxiety/depression–sleep disorder common module. Berberine showed significant proximity to the anxiety/depression-related module. In view of the reported anxiolytic and sleep-regulatory effects of genistein [15,16] and the reported effects of berberine on drug withdrawal-associated emotional disturbances [17,18], these compounds may be considered candidate active constituents of JYF that warrant further investigation. Previous studies have also suggested potential antidepressant activity of jatrorrhizine [19]. However, direct experimental evidence supporting the effects of jatrorrhizine and columbamine on benzodiazepine withdrawal syndrome remains limited. These compounds should therefore be regarded as candidate active constituents rather than established pharmacological mediators. Collectively, these findings suggest that the effects of JYF on withdrawal-associated emotional and sleep-related abnormalities may be associated with coordinated interactions among multiple constituents and molecular targets. However, network proximity analysis reflects topological association rather than direct pharmacological causality. The specific pharmacological contributions of genistein, berberine, columbamine, and jatrorrhizine, as well as their links to specific metabolic or monoaminergic pathways, require further experimental validation.

Widely targeted metabolomics revealed marked alterations in arachidonic acid, DHA, DPA, and several long-chain acylcarnitines after diazepam withdrawal. Acylcarnitines mediate the transport of long-chain fatty acids into mitochondria for β-oxidation, and their dysregulation may therefore reflect changes in fatty acid transport and mitochondrial oxidative metabolism [20]. Arachidonic acid and DHA are major constituents of neural membrane phospholipids and are involved in synaptic function and lipid-mediated inflammatory signaling [21]. Taken together, these findings are consistent with disturbances in membrane lipid homeostasis, fatty acid utilization, and mitochondrial energy metabolism during diazepam withdrawal. Several metabolites also showed changes associated with JYF intervention. AMP, adrenic acid, and DPA were altered after withdrawal and shifted toward control levels following JYF administration. AMP is closely related to cellular energy status and AMPK-mediated energy sensing [22]. Adrenic acid participates in neural membrane remodeling and oxidative lipid metabolism [23,24], whereas DPA-derived lipid mediators have been implicated in the resolution of neuroinflammation [25]. In addition, 2-oxoglutarate links the TCA cycle with glutamate/glutamine metabolism and mitochondrial energy homeostasis [26]. The observed correlations of AMP, adrenic acid, and DPA with CORT and OXA further suggest an association between neuroendocrine changes and metabolic remodeling. However, these correlations do not establish causal relationships. These metabolites may therefore be considered candidate pharmacodynamic metabolic markers associated with the response to JYF.

Diazepam withdrawal was also accompanied by marked disturbances in amino acid and monoamine neurotransmitter metabolism. Glutamate, GABA, aspartate, and glycine were all reduced, indicating disruption of excitatory and inhibitory amino acid neurotransmitter homeostasis. Previous studies have shown that chronic benzodiazepine exposure and withdrawal can induce neuroadaptive changes in glutamic acid decarboxylase and GluR1-containing AMPA receptors, thereby affecting GABAergic and glutamatergic neurotransmission [27,28]. Tryptophan metabolism was also altered. One study reported reduced midbrain tryptophan, together with region-dependent changes in serotonin and 5-hydroxyindoleacetic acid, after 22 days of diazepam administration followed by 48 h of withdrawal [29]. By contrast, Andrews et al. observed increased serotonin in the cortex and hippocampus after 21 days of diazepam treatment followed by 24 h of withdrawal [30]. These findings suggest that serotonergic responses may vary according to withdrawal duration and brain region. In the present study, midbrain serotonin was increased after diazepam withdrawal, whereas JYF shifted serotonin toward control levels and was associated with changes in 5-hydroxytryptophan and several kynurenine-pathway metabolites. Catecholaminergic metabolism showed a similarly variable pattern. Previous work reported reductions in dopamine and norepinephrine across several brain regions after prolonged diazepam exposure and withdrawal, together with increased striatal HVA [31]. In our study, dopamine was likewise reduced, whereas norepinephrine was increased. These differences may reflect variation in exposure duration, withdrawal stage, or brain region. JYF shifted both dopamine and norepinephrine toward control levels. These findings suggest that JYF is associated with modulation of withdrawal-related catecholaminergic disturbances, although the underlying mechanisms require further validation.

Integrated analysis of differential metabolites and candidate JYF targets further suggested that amino acid metabolism and monoamine neurotransmitter metabolism are important processes associated with diazepam withdrawal and JYF intervention. Tryptophan, phenylalanine, and tyrosine serve not only as substrates for protein synthesis but also as precursors for the biosynthesis of 5-HT and catecholamine neurotransmitters. Consistent with this relationship, targeted quantitative analysis showed alterations in tryptophan, phenylalanine, tyrosine, 5-HT, dopamine, and norepinephrine that were in agreement with the pathway enrichment results. Further constituent–target–pathway analysis linked berberine and genistein to tryptophan- and aromatic amino acid-related metabolic pathways through candidate targets including *IDO1*, *TDO2*, *MAOA*, and *MAOB*. These associations suggest that the two compounds may be involved in JYF-related modulation of monoamine neurotransmitter metabolism. Previous studies have shown that berberine can downregulate hippocampal *IDO1* and *MAOA* expression and ameliorate abnormalities in the kynurenine/serotonin pathway [32]. Genistein has also been reported to inhibit MAO-A and MAO-B activity and to regulate CRF and serotonin levels, with accompanying improvements in sleep architecture in models of stress-induced sleep disturbance [33,34]. These findings provide biological plausibility for the constituent–target–pathway associations identified in the present study. However, these network-based relationships remain associative, and the specific contributions of berberine, genistein, and the related candidate targets to the peri-withdrawal effects of JYF require direct experimental validation.

In summary, this study provides converging evidence for the effects of JYF during diazepam withdrawal across multiple levels, including behavioral and EEG phenotypes, in vivo constituent exposure, network topology, and brain metabolic alterations. However, several limitations should be acknowledged. The present model used abrupt diazepam discontinuation and included only male animals, whereas benzodiazepine withdrawal in clinical practice is generally managed through gradual tapering. The clinical relevance and applicable population of JYF therefore remain to be established. In addition, the current design evaluated JYF as a peri-withdrawal intervention rather than as a treatment initiated after withdrawal symptoms had become established. The mechanistic interpretation should also remain cautious. Brain constituent identification, network proximity analysis, joint pathway enrichment, steady-state metabolite quantification, and correlation analysis provide primarily qualitative, topological, or associative evidence. This limitation is further reinforced by the use of different species for constituent exposure and pharmacodynamic studies and by the integration of experimental measurements with network-based predictions. Future studies should therefore assess quantitative tissue exposure in the same species used for pharmacodynamic experiments, incorporate metabolic flux analysis, evaluate the expression and activity of key enzymes, and apply targeted interventions to the HPA axis and orexin-related pathways. These approaches will be important for defining the candidate active constituents of JYF and for clarifying the mechanisms associated with its peri-withdrawal effects.

## 4. Materials and Methods

### 4.1. Chemicals and Reagents

Diazepam injection (Lot No. 2206181) was obtained from Tianjin Jinyao Pharmaceutical Co., Ltd. (Tianjin, China). The c-Fos monoclonal antibody (mouse IgG1; Cat. No. 66590-1-Ig) was purchased from Proteintech (Wuhan, China). The CORT assay kit (Lot No. 20240510) was obtained from Wuhan Beiyinlai Biotechnology Co., Ltd. (Wuhan, China), and the mouse orexin A assay kit (Lot No. GR20240910) was obtained from Geneme Biotechnology Co., Ltd. (Shanghai, China). Isoflurane (Lot No. 2024070701) was purchased from RWD Life Science Co., Ltd. (Shenzhen, China). A 4% tissue/cell fixative solution was obtained from Solarbio Science & Technology Co., Ltd. (Beijing, China). Hematoxylin staining solution (Cat. No. P0064) and an HRP-conjugated goat anti-mouse IgG secondary antibody (Cat. No. PN0080) were purchased from Wuhan Pinofei Biotechnology Co., Ltd. (Wuhan, China). HPLC-grade methanol, acetonitrile, and formic acid were purchased from Dikma Technologies Inc. (Beijing, China). Ultrapure water was generated using a Milli-Q purification system (Millipore, Burlington, MA, USA).

### 4.2. Instrumentation and Equipment

The HPLC–HRMS system used for constituent identification consisted of an Agilent 1200 liquid chromatograph coupled with a 6520 Q-TOF mass spectrometer, with data acquisition and processing performed using MassHunter 10.0 software (Agilent Technologies, Santa Clara, CA, USA). Metabolomic analyses were conducted using an ACQUITY H-Class liquid chromatograph coupled with a Xevo TQ-S micro mass spectrometer and operated with MassLynx V4.1 software (Waters Corporation, Milford, MA, USA). Targeted HPLC–MS/MS analysis was performed on an ExionLC-20AC system coupled with a SCIEX 6500+ triple-quadrupole mass spectrometer equipped with an IonDrive™ Turbo V ion source. Data acquisition and processing were performed using Analyst 1.7 and MultiQuant 3.0.3 software, respectively (AB SCIEX, Framingham, MA, USA). The DigBeh-LM4 automated open-field video analysis system and the JLBehv-BWM-4 light–dark box video analysis system were purchased from Shanghai Jiliang Software Technology Co., Ltd. (Shanghai, China). A laboratory-developed small-animal EEG monitoring system (Patent No. 2026206496066) was used for electrophysiological recordings. This system included a shielded chamber equipped with circadian rhythm control, sound insulation, and antistatic functions, freely moving animal cages, and an AD PowerLab 8/35 multichannel electrophysiological recording system (AD Instruments, Sydney, Australia). A 71,000 automated stereotaxic instrument, a 78,001 miniature handheld cranial drill, an R580 animal anesthesia machine, and a ThermoStar body-temperature maintenance system were purchased from RWD Life Science Co., Ltd. (Shenzhen, China). EEG and EMG electrode chips were fabricated in-house. A QUICK 204 soldering iron (QUICK Intelligent Equipment Co., Ltd., Changzhou, China) and silver wire (A-M Systems, Sequim, WA, USA) were used for electrode preparation. Laboratory equipment included a Targin VX-III multi-tube vortex mixer (Beijing Tajin Technology Co., Ltd., Beijing, China), G12402031 electric tissue homogenizer (GINGKO, Beijing, China), AMR-100 microplate reader (Hangzhou Allsheng Instruments Co., Ltd., Hangzhou, China), GNP-9080 water-jacketed constant-temperature incubator (Shanghai Jinghong Laboratory Instrument Co., Ltd., Shanghai, China), and Rotanta 460R high-speed refrigerated centrifuge (Hettich, Tuttlingen, Germany). Ultrapure water was produced using a Milli-Q Integral 3 purification system (Millipore, Burlington, MA, USA).

### 4.3. Preparation of the JYF Aqueous Extract

The JYF formula consisted of 12 medicinal herbs: *Panax quinquefolius* (15 g), bran-processed *Atractylodes macrocephala* (10 g), raw *Astragalus membranaceus* (10 g), *Bupleurum chinense* (15 g), *Angelica sinensis* (6 g), *Phellodendron chinense* (12 g), *Aucklandia lappa* (6 g), *Citrus reticulata* (6 g), *Cimicifuga foetida* (5 g), *Gastrodia elata* (6 g), *Glycyrrhiza uralensis* (6 g), and *Aquilaria sinensis* (6 g). All herbal materials were purchased from Beijing Tongrentang Co., Ltd. (Beijing, China). The production, processing, and quality control procedures for the herbal materials complied with the requirements of the *Chinese Pharmacopoeia* (2020 edition) and the *Beijing Processing Specifications for Chinese Herbal Pieces* (2023 edition). A ten-fold amount of the prescribed JYF formula (1.03 kg) was subjected to standardized extraction according to the *Management Specifications for Decoction Rooms of Medical Institutions*. *Panax quinquefolius* was decocted separately, whereas the remaining herbs were soaked in 10 volumes of water for 2 h and decocted twice, with each decoction lasting 30 min under gentle boiling conditions. The resulting decoctions were filtered, and the filtrates were combined with the separately prepared *Panax quinquefolius* decoction. The combined extract was concentrated under reduced pressure using a rotary evaporator at 80 °C to obtain a final concentration of 1.8 g/mL (crude herb equivalent). The prepared JYF extract was stored at −80 °C until further use.

### 4.4. Network Medicine Analysis of JYF

#### 4.4.1. Collection and Validation of Susceptibility and Regulatory Genes Associated with Diazepam Withdrawal and Related Symptoms

Standardized search terms were defined using Medical Subject Headings and the Unified Medical Language System to collect genes associated with diazepam withdrawal and related symptoms. Withdrawal- and dependence-related terms included “Withdrawal Syndrome”, “Substance Withdrawal Syndrome”, “Rebound Insomnia”, “Drug Withdrawal”, “Drug Dependence”, “Dependence”, “Withdrawal”, and “Hypnotic Dependence”. Anxiety/depression-related terms included “Depression” and “Anxiety”, whereas sleep-related terms included “Sleep Wake Disorder”, “Insomnia”, “Circadian Rhythm Sleep Disorders”, and “Sleep Disorder”.

These terms were used to search GeneCards and Online Mendelian Inheritance in Man (OMIM). GeneCards entries with a Relevance score > 5 were retained, while all genes explicitly associated with the corresponding phenotype in OMIM were included. Genes obtained from different terms and databases were merged, restricted to Homo sapiens, standardized to HGNC-approved gene symbols, and deduplicated. Entries that could not be mapped to standard human gene symbols were removed.

After standardization and deduplication, the W, AD, and S gene sets were constructed separately. Pairwise and three-way intersection analyses were then used to derive the WAD, WS, and WADS modules. These six disease/symptom modules were used for subsequent network proximity analysis. Genes in each module were mapped to the human PPI network, and the original gene-set size, number of successfully mapped genes, and characteristics of the largest connected component were recorded.

#### 4.4.2. Collection and Curation of JYF Constituent Targets

The circulating JYF constituents identified in Section 4.3 were queried against ChEMBL. Compound names, structures, and ChEMBL compound IDs were manually verified, with free parent compounds prioritized. Based on a previous study [35], activity records were retained when the target was a human single protein, Ki, Kd, IC50, or EC50 was ≤10 μM, and the relation operator was “=”, “<”, or “≤”. Single-concentration percentage-activity records were retained only when the test concentration was ≤10 μM and the effect was ≥50%. Records involving inconsistent structures or IDs, cytotoxicity assays, ADME data, non-single-protein targets, or unclear activity evidence were excluded. Targets were standardized to HGNC-approved gene symbols and deduplicated at the constituent–target level before network proximity analysis.

#### 4.4.3. Calculation of Network Proximity

Network proximity analysis was used to quantify the topological relationship between targets of circulating JYF constituents and the disease/symptom modules according to Guney et al. [36]. A human PPI network was constructed from physical protein–protein interactions in STRING v12.0, retaining interactions with a combined score ≥ 700. After gene-symbol standardization and removal of self-loops and duplicate edges, the largest connected component was used as the background network G. Let T denote the JYF target set and S the disease/symptom gene module. Network distance was calculated using the following equation.

$$d\left(T,S\right)=\frac{1}{\left|T\right|}\sum_{t\in T}\underset{s\in S}{\min}\;d_{G}\left(t,s\right)$$

where *d_G_*(*t*, *s*) denotes the shortest-path length between drug target *t* and disease-associated gene *s* in network *G*, and |*T*| denotes the number of drug targets successfully mapped to the PPI network.

To account for node-degree bias, network nodes were stratified into degree bins. Degree- and set-size-matched random target and disease sets were then generated for *T* and *S*, respectively, using 1000 permutations. *Z* scores were calculated from the resulting null distribution of randomized network distances.

$$Z=\frac{d_{obs}-\mu_{R}}{\sigma_{R}}$$

where *d*_obs_ is the observed network distance, and *μ_R_* and *σ_R_* are the mean and standard deviation, respectively, of the randomized network distances. The one-sided empirical *p* value was defined as the proportion of permutations in which the randomized distance was less than or equal to the observed distance. For ease of interpretation, −*Z* was defined as the network proximity score, with larger positive values indicating stronger topological proximity.

Network proximity was calculated between the pooled target set of circulating JYF constituents, as well as the targets of 13 individual circulating constituents, and the six disease modules. Significant proximity was defined as *Z* < 0 and *p* < 0.05, and multiple testing was corrected using the Benjamini–Hochberg method. All analyses were performed in R version 4.4.1, and igraph version 2.2.3 was used to construct the network and calculate unweighted shortest paths.

### 4.5. Experimental Animals

Male C57BL/6J mice and male Sprague–Dawley rats, both 6–7 weeks old and of specific pathogen-free (SPF) grade, were purchased from Beijing HFK Bioscience Co., Ltd. (Beijing, China; license No. SCXK (Beijing) 2019-0008). All animals were housed in individually ventilated cages (461 mm × 274 mm × 229 mm) at approximately 22 °C and 50% relative humidity, acclimatized for 1 week before the experiments, and provided with free access to water and commercial chow. At the end of the experiments, all animals were euthanized. The animal experimental protocol was approved by the Animal Ethics Committee of the Medical Experimental Center, China Academy of Chinese Medical Sciences (No. ERCCACMS21-2303-05).

### 4.6. Pharmacodynamic Study of JYF

#### 4.6.1. Animal Grouping, Modeling, and Administration

Sixty-five mice were randomly assigned using a random-number table to five groups: control (*n* = 15), model (*n* = 15), JYF low dose (JYF-L; *n* = 10), JYF medium dose (JYF-M; *n* = 10), and JYF high dose (JYF-H; *n* = 15). Control mice received normal saline subcutaneously, whereas all other groups received diazepam (12 mg/kg, s.c.) [37] once daily at approximately 10:30 for 28 consecutive days. Diazepam was then discontinued to induce spontaneous withdrawal.

JYF administration began 4 days before diazepam discontinuation. JYF was given by oral gavage 30 min before each diazepam injection and was continued through post-withdrawal day 3, for a total of 7 days. Based on conversion of the clinical human dose to the mouse-equivalent dose, JYF was administered at 7.8, 15.5, and 31.0 g/kg in the JYF-L, JYF-M, and JYF-H groups, respectively. Control and model mice received an equal volume of distilled water by gavage. Five mice from each of the control, model, and JYF-H groups were selected for EEG/EMG monitoring and sleep analysis.

#### 4.6.2. Assessment of General Physiological Status

The general condition, average food intake, and water intake of mice in each group were observed and recorded during the withdrawal period. Body weight was recorded before model establishment and after the final administration.

#### 4.6.3. Recording and Analysis of Sleep–Wake Behavior

Sleep–wake behavior was continuously recorded for 72 h after diazepam withdrawal to evaluate withdrawal-associated sleep disturbances and the effects of JYF.

##### Electrode Implantation and EEG/EMG Recording

EEG/EMG electrodes were implanted 7 days before the start of recording to allow sufficient postoperative recovery and adaptation. Mice were anesthetized with isoflurane (4% for induction and 2% for maintenance), and the head was fixed in a stereotaxic apparatus. After shaving and disinfection with 75% ethanol, a longitudinal midline incision was made in the scalp to expose the skull. Three burr holes (0.8 mm in diameter) were drilled at 1.0 mm anterior to bregma and 1.5 mm lateral on both sides, and at 1.5 mm anterior to lambda and 1.5 mm lateral to the left, taking care not to penetrate the meninges. Screws connected to an electrode chip containing six pins arranged in a 2 × 3 configuration were implanted for EEG recording. Two silver wires were inserted into the posterior neck muscles and connected to the electrode chip for EMG recording. The screws and electrode chip were secured with dental cement. After surgery, each mouse received penicillin (40,000 units) once daily for 3 days to prevent infection. Mice were housed individually in shielded cages and acclimatized to a light–dark cycle with lights on from 07:00 to 19:00 and lights off from 19:00 to 07:00 the following day. At the start of formal recording (09:00 on post-withdrawal day 1), the electrodes were connected to a multichannel physiological recording system, and EEG and EMG signals were continuously recorded for 72 h over the first 3 days after withdrawal. During the recording period, distilled water or JYF was administered by oral gavage at approximately 10:00 each day.

##### Sleep–Wake State Scoring and Analysis

According to the method described by Zhang et al. [38], sleep recordings were analyzed offline using Sirenia^®^ Sleep software Version 1.8.0 (Pinnacle Technology Inc., Lawrence, KS, USA). EEG and EMG signals were amplified and band-pass filtered at 0.5–30 Hz and 20–200 Hz, respectively. Wakefulness was characterized by high-frequency, low-amplitude desynchronized EEG activity accompanied by relatively high EMG activity. NREM sleep was characterized by high-amplitude, low-frequency EEG slow wave activity, with δ wave activity at least 50% greater than that observed during wakefulness, together with reduced EMG tone. REM sleep was characterized by high-frequency, low-amplitude EEG activity accompanied by EMG atonia. Wakefulness, NREM sleep, and REM sleep were automatically scored in 10 s epochs, and the classifications were subsequently verified manually.

Abnormal spike-wave discharges (SWDs) were identified and quantified using a threshold-based approach. EEG recordings were analyzed offline against the normal background EEG activity. SWDs were defined as high-amplitude spike components followed by slow waves that were clearly distinguishable from background EEG activity and artifacts. Spike Histogram parameters were applied uniformly across all recordings as follows: detection threshold, 0.3 mV; total trigger interval, 300 ms; and pretrigger interval, 100 ms. Candidate events were reviewed in the Spike Histogram discriminator view generated using the “Analyze Selection” function to confirm true events and exclude artifacts. Confirmed SWDs were then quantified using the rate meter. The same detection parameters were applied to all EEG recordings to ensure consistency and reproducibility.

#### 4.6.4. Open Field and Light–Dark Box Tests

Following the final administration, the open-field test and light–dark box test were performed to assess spontaneous locomotor activity, exploratory behavior, and anxiety-like behavior.

The open-field apparatus consisted of a waterproof square arena measuring 50 cm × 50 cm, with the floor divided into 16 equal squares; the central four squares were defined as the central zone, and the remaining 12 squares as the peripheral zone. Each mouse was gently placed in the center of the arena, and an automated open-field video analysis system was used to record total movement distance, time spent in the central zone, number of entries into the central zone, and other parameters over 5 min.

The light–dark box apparatus consisted of an illuminated chamber and a dark chamber connected by a shuttle passage. Each mouse was placed in the illuminated chamber with its back facing the passage, and a light–dark box video analysis system was used to record the latency to first entry into the dark chamber, time spent in the dark chamber, and number of transitions between the two chambers over 5 min.

After each mouse was tested, the inner walls and floor of the apparatus were wiped with 75% ethanol to remove residual odors and minimize interference with subsequent tests. All behavioral tests were conducted in a quiet environment under constant temperature and humidity.

### 4.7. Histopathological and Immunohistochemical Analysis of Brain Tissue

After euthanasia, the brains were collected, and the midbrain tissue was dissected and fixed in 4% PFA for 24 h. Following fixation, the tissues were dehydrated through a graded ethanol series, cleared in xylene, infiltrated with paraffin, and embedded in paraffin blocks. Serial coronal sections of 4–5 μm thickness were subsequently prepared using a microtome.

For H&E staining, sections were deparaffinized and rehydrated, stained with hematoxylin for 5 min, differentiated in acid alcohol, and counterstained with eosin for 2 min. The sections were then dehydrated through graded ethanol, cleared in xylene, and mounted with neutral resin. Finally, the sections were examined and imaged under a light microscope.

For c-Fos staining, midbrain coronal sections were routinely deparaffinized and rehydrated, followed by washing with PBS. Heat-induced antigen retrieval was performed in citrate buffer, followed by incubation with 3% hydrogen peroxide to block endogenous peroxidase activity. After washing with PBS, the tissue area was outlined with a hydrophobic barrier pen, and normal goat serum blocking solution was applied for 30 min at 37 °C. After removal of the blocking serum, the sections were incubated with a c-Fos primary antibody (1:100) overnight at 4 °C in a humidified chamber. The following day, after thorough washing with PBS, the sections were incubated with an HRP-conjugated goat anti-mouse IgG secondary antibody (1:1000) for 1 h at room temperature. After PBS washing, DAB working solution was added for chromogenic development, which was monitored microscopically and terminated at the appropriate time. The sections were subsequently counterstained with hematoxylin for 3–5 min, differentiated in acid alcohol for several seconds, and rinsed under running water for bluing. After dehydration through graded ethanol, clearing with n-butanol and xylene, and mounting with neutral resin, the sections were examined and imaged under a light microscope.

### 4.8. Measurement of Serum CORT and Serum and Brain OXA Levels

Blood samples were collected from the retro-orbital plexus of mice and allowed to stand at room temperature for 4 h, followed by centrifugation at 4000× *g* for 15 min at 4 °C to separate the serum. An appropriate amount of brain tissue was homogenized thoroughly in precooled tissue homogenization buffer, and the supernatant was collected after centrifugation. Serum CORT and OXA levels and brain OXA levels were determined using the corresponding ELISA kits according to the manufacturers’ instructions.

### 4.9. Identification of Chemical Constituents of JYF and Its Serum and Brain-Exposed Constituents

#### 4.9.1. Sample Preparation

A 0.20 mL aliquot of JYF extract was mixed with 1.00 mL of 70% methanol, vortexed, and centrifuged at 20,000× *g* for 10 min. The supernatant was collected as the JYF test solution. Six rats received a single oral dose of JYF extract (31 g/kg). Blood samples were collected before dosing and 2 h after dosing, and brain tissues were collected 2 h after dosing. Serum was separated from the blood samples. Serum samples from the six animals were pooled, and a 300 μL aliquot was mixed with 900 μL methanol, vortexed, and centrifuged at 20,000× *g* for 10 min. The supernatant was evaporated to dryness under nitrogen and reconstituted in 100 μL methanol to obtain the serum sample solution. Brain tissues from the six animals were pooled and homogenized in 5 mL ultrapure water. A 300 μL aliquot of the homogenate was mixed with 900 μL methanol, vortexed, and centrifuged at 20,000× *g* for 10 min. The supernatant was dried under nitrogen and reconstituted in 100 μL methanol to obtain the brain sample solution.

#### 4.9.2. Chromatographic Conditions

Chromatographic separation was performed using an HSS T3 column (1.8 μm, 2.1 mm × 100 mm; Waters Corporation, Milford, MA, USA). The mobile phase consisted of 0.1% formic acid in water (A) and 0.1% formic acid in acetonitrile (B). The gradient elution program was as follows: 0–25 min, 5–25% B; 25–30 min, 25–31% B; 30–60 min, 31–45% B; 60–65 min, 45–100% B; and 65–70 min, 100% B, followed by a 3 min post-run period. The flow rate was 0.3 mL/min, the column temperature was 30 °C, the autosampler temperature was 4 °C, and the injection volume was 1 μL.

#### 4.9.3. Mass Spectrometry Conditions

Electrospray ionization (ESI) was used in both positive and negative ion modes. The nebulizer pressure was 10 psi, the gas temperature was 300 °C, and the gas flow rate was 10 L/min. The capillary voltage was 3500 V and the fragmentor voltage was 175 V. The MS1 and MS2 scanning ranges were *m*/*z* 50–1500 and *m*/*z* 20–1500, respectively. The slope of the linear collision-energy ramp was set to 5.

#### 4.9.4. Data Acquisition and Constituent Identification

Total ion chromatograms of the JYF aqueous extract and serum and brain samples collected after oral administration were acquired under the chromatographic and mass spectrometric conditions described above. Raw data were processed in MassHunter to obtain retention times, mass-to-charge ratios, peak areas, and related information for the major ion peaks. Molecular formulas were preliminarily assigned from accurate mass measurements using a mass-error tolerance of <10 ppm. Constituents were then identified from MS/MS fragment ions and fragmentation patterns in combination with an in-house TCM reference-standard library and the published literature.

### 4.10. Brain Tissue Metabolomics Analysis

After completion of the behavioral tests, blood samples were collected from the retro-orbital venous plexus of mice in the control, model, and JYF-M groups. The mice were then euthanized by cervical dislocation, and brain tissues were rapidly collected. Midbrain tissues were isolated, accurately weighed, mixed with precooled saline at a tissue-to-solvent ratio of 1:4 (*w*/*v*), and homogenized on ice. A 100 μL aliquot of the homogenate was mixed with three volumes of acetonitrile, vortexed for 5 min, and centrifuged at 20,000× *g* for 5 min at 4 °C. The supernatant was collected for widely targeted metabolomics and targeted neurotransmitter analysis.

#### 4.10.1. Sample Preparation

Samples for widely targeted metabolomics were prepared according to a previously reported method [39]. A 30 μL aliquot of supernatant was mixed with 20 μL of derivatization reagent containing 200 mM 3-NPH in 75% methanol and 96 mM EDC–6% pyridine in methanol. The mixture was incubated at 30 °C for 60 min, and the reaction was terminated by adding 350 μL of ice-cold 50% methanol. After centrifugation at 4000× *g* for 30 min at 4 °C, the supernatant was collected for LC–MS/MS analysis. A 10 μL aliquot from each sample was pooled to prepare the QC sample.

Samples for targeted neurotransmitter analysis were prepared according to a previously reported method [40]. A 10 μL aliquot of supernatant was mixed with 10 μL of 100 mM sodium borate and 10 μL of 1% benzoyl chloride in acetonitrile, incubated at 25 °C for 5 min, and centrifuged at 20,000× *g* for 10 min at 25 °C. A 24 μL aliquot of the derivatized sample was mixed with 6 μL of internal standard mixture prepared by derivatizing mixed reference standards (100 ng/mL) with d5-benzoyl chloride, followed by LC–MS/MS analysis.

#### 4.10.2. Widely Targeted Metabolomics Analysis Conditions

Chromatographic Conditions: Separation was performed on a BEH C18 column (2.1 mm × 100 mm, 1.7 μm; Waters Corporation, Milford, MA, USA). The mobile phase consisted of 0.1% formic acid in water (A) and acetonitrile:isopropanol (7:3, *v*/*v*) (B). The gradient was as follows: 0–1 min, 5% B; 1–5 min, 5–30% B; 5–9 min, 30–50% B; 9–12 min, 50–79% B; 12–15 min, 78–95% B; 15–16 min, 95–100% B; 16–18 min, 100% B; 18–18.1 min, 100–5% B; and 18.1–20 min, 5% B. The flow rate was 0.4 mL/min, the column temperature was 40 °C, the autosampler temperature was 10 °C, and the injection volume was 5 μL.

Mass Spectrometry Conditions: ESI was operated in positive- and negative-ion modes with capillary voltages of 3200 and −1200 V, respectively. The desolvation temperature was 550 °C, the cone voltage was 30 V, the desolvation gas flow was 1000 L/h, and the source temperature was 150 °C. Nitrogen was used as the desolvation gas. MRM was performed for semi-quantitative profiling of over 200 metabolites across multiple chemical classes, including carbohydrates, organic acids, carnitines, fatty acids, and bile acids. Ion-transition parameters and data processing procedures followed the previously reported method [39].

#### 4.10.3. Targeted Neurotransmitter and Related Metabolite Analysis Conditions

Chromatographic Conditions: Separation was performed on a PFP C18 column (2.1 mm × 50 mm, 1.8 μm; Waters, USA). The mobile phase consisted of water containing 5 mM ammonium formate and 0.1% formic acid (A) and methanol containing 0.1% formic acid (B). The gradient was as follows: 0–1.0 min, 20% B; 1.0–2.0 min, 20–50% B; 2.0–6.0 min, 50–70% B; 6.0–6.5 min, 70–95% B; 6.5–8.0 min, 95% B; 8.0–8.1 min, 95–20% B; and 8.1–10.0 min, 20% B. The flow rate was 0.3 mL/min, the column temperature was 35 °C, the autosampler temperature was 4 °C, and the injection volume was 2 μL.

Mass Spectrometry Conditions: ESI was performed with curtain gas at 35 psi, collision gas at 9 psi, and a spray voltage of 5500 V. The source temperature was maintained at 550 °C, and Ion Source Gas 1 and Ion Source Gas 2 were each set at 55 psi. Positive-ion MRM was used for the quantitative analysis of 40 neurotransmitters and related metabolites. Authentic reference standards corresponding to the 40 neurotransmitters and related metabolites were used for metabolite identification and calibration. A chemical derivatization-based pretreatment strategy was applied to improve chromatographic retention and enhance mass spectrometric sensitivity. Calibration curves were established using solvent-based standard solutions rather than matrix-matched calibration standards. d5-benzoyl chloride-based isotope labeling was used for internal correction, and derivatized standards and samples were analyzed under identical LC–MS/MS conditions. Quantification was performed based on the corresponding calibration curves and isotope-labeling-based correction. Ion-transition parameters and analytical procedures followed the previously published method [40].

#### 4.10.4. Metabolomics Data Preprocessing and Differential Metabolite Analysis

Raw data were processed using MassLynx 4.1 and MultiQuant 3.0.3. Data processing included chromatographic peak identification, peak-area integration, and metabolite annotation, followed by merging of the results obtained from positive- and negative-ion modes. One QC sample was injected after every 10 study samples throughout the analytical sequence to monitor analytical stability. Metabolite abundances were evaluated semi-quantitatively based on relative peak intensity (peak area), and the processed data were used to generate the sample-by-variable matrix for subsequent multivariate statistical analysis.

PCA was performed to evaluate overall metabolic variation and sample clustering patterns. OPLS-DA was performed to assess group discrimination among experimental groups. The quality and predictive performance of OPLS-DA models were evaluated using R^2^Y and Q^2^ values, and permutation testing was performed to evaluate model robustness and potential overfitting. Differential metabolites were identified according to predefined criteria of VIP > 1, *p* < 0.05, and FC > 1.20 or FC < 0.83. Pathway enrichment analysis was performed based on the identified differential metabolites. Differential metabolite screening, annotation, and pathway analysis were conducted using OmicShare online platform (OmicShare, Guangzhou, China; https://www.omicshare.com/, accessed on 17 August 2026), and MetaboAnalyst 6.0.

### 4.11. Statistical Analysis

Statistical analyses and graphical presentation were performed using SPSS 20.0 and GraphPad Prism 9.5. Data are presented as mean ± standard error of the mean (SEM). For two-group comparisons, normally distributed data with homogeneous variances were analyzed using an independent-samples *t*-test. Non-repeated measurements involving multiple groups were analyzed by one-way analysis of variance (ANOVA), followed by Dunnett’s post hoc test; when the assumption of homogeneity of variance was not met, Tamhane’s T2 test was used. Repeated-measures data were analyzed using two-way repeated-measures ANOVA to assess group and time effects. All tests were two-sided, and *p* < 0.05 was considered statistically significant.

## 5. Conclusions

By establishing a mouse model of spontaneous withdrawal following long-term diazepam exposure and combining behavioral assessment with EEG/EMG monitoring, this study showed that peri-withdrawal JYF intervention attenuated anxiety-related behavioral abnormalities, sleep architecture disturbances, and abnormal EEG activity, while reducing serum CORT and serum and brain OXA levels. JYF intervention was also associated with changes in brain lipid and energy metabolism, as well as tryptophan- and tyrosine-related metabolic pathways. These effects may be associated with modulation of cerebral monoamine neurotransmitter homeostasis, including 5-HT, DA, and NE. Genistein and berberine were identified as candidate active constituents that warrant further experimental validation. Overall, these findings provide preclinical evidence for the effects of JYF during diazepam withdrawal; however, its clinical relevance remains to be established in future studies using designs that more closely reflect clinical benzodiazepine tapering.

## Figures and Tables

**Table 1:** Chemical Constituents Identified in JYF and in Rat Serum and Brain Tissue Following Oral Administration of JYF.

NO	t_R_ (min)	Compound	Formula	Ion Adduct	Observed	Theoretical	MS2 Fragments	Error (ppm)	Extract	Serum	Brain
1	2.586	Gastrodin *	C_13_H_18_O_7_	[M + COOH]^−^	331.1048	331.1035	123.0449, 44.9985	4.70	+	+	+
2	2.735	Gallic acid *	C_7_H_6_O_5_	[M − H]^−^	169.0145	169.0142	125.0102	1.49	+	+	+
3	4.833	p-Hydroxybenzyl alcohol *	C_7_H_8_O_2_	[M − H]^−^	123.0457	123.0452	105.0342, 77.0401	4.41	+	+	+
4	9.537	Magnocurarine [1]	C_19_H_24_NO_3_	[M]^+^	314.1756	314.1751	269.1162, 107.0486	1.69	+	+	+
							175.0746, 58.0660				
5	9.831	Lupalbigenin/Licocoumarone	C_21_H_20_O_6_	[M − H]^−^	367.1191	367.1187	193.0540, 273.0463	1.05	+	—	—
							134.0374, 217.0344				
6	11.18	Phellodendrine [1]	C_20_H_24_NO_4_	[M]^+^	342.173	342.1705	192.1015, 177.0780	8.81	+	+	+
7	12.679	Magnoflorine [1]	C_20_H_24_NO_4_	[M]^+^	342.1707	342.1705	297.1117, 282.0879, 265.0858, 237.0909	2.09	+	+	+
							58.0661				
8	13.829	Lupalbigenin/Licocoumarone	C_21_H_20_O_6_	[M − H]^−^	367.1197	367.1187	191.0565, 273.0467	2.68	+	—	—
							155.0347, 234.0368				
9	14.582	Lotusine [1]	C_19_H_24_NO_3_	[M]^+^	314.1766	314.1751	269.1170, 237.0908, 175.0750, 107.0491	4.87	+	+	+
10	15.19	Agarotetrol *	C_17_H_18_O_6_	[M + H]^+^	319.118	319.1176	301.1066, 283.0954	1.21	+	+	+
							255.1008, 264.0452				
11	16.729	Ferulic acid *	C_10_H_10_O_4_	[M − H]^−^	193.0514	193.0506	178.0276, 134.0369	3.96	+	+	+
12	16.982	Calycosin-7-O-β-D-glucoside *	C_22_H_22_O_10_	[M + COOH]^−^	491.1232	491.1195	430.0946, 283.0632, 268.0387, 44.9992	8.29	+	+	+
13	17.629	Liquiritin *	C_21_H_22_O_9_	[M − H]^−^	417.1204	417.1191	255.0698, 235.0091,	3.1	+	+	+
							119.0503, 91.0195				
14	17.681	Rutin *	C_27_H_30_O_16_	[M − H]^−^	609.1487	609.1461	300.0302, 151.0040				
15	17.878	Isoferulic acid *	C_10_H_10_O_4_	[M − H]^−^	193.0514	193.0506	178.0270, 134.0369	3.96	+	+	+
16	19.983	Narirutin [2]	C_27_H_32_O_14_	[M + H]^+^	581.1891	581.1878	273.0757, 195.0283, 153.0179, 85.0288, 71.0497	5.29	+	+	+
17	20.98	Naringin *	C_27_H_32_O_14_	[M − H]^−^	579.1751	579.1719	459.1202, 271.0639,	5.47	+	—	—
							151.0038				
18	21.728	Hesperidin *	C_28_H_34_O_15_	[M − H]^−^	609.1871	609.1825	301.0740, 264.0118	7.55	+	+	+
19	22.734	Columbamine [1]	C_20_H_20_NO_4_	[M]^+^	338.1389	338.1387	323.1146, 308.0918, 294.1119, 280.0945	0.64	+	+	+
20	22.828	Neohesperidin *	C_28_H_34_O_15_	[M − H]^−^	609.1855	609.1825	301.0729, 264.0113	4.93	+	—	—
21	23.233	Jatrorrhizine [1]	C_20_H_20_NO_4_	[M]^+^	338.1391	338.1392	323.1142, 308.0898, 294.1115, 280.0916	1.52	+	+	+
22	23.826	Isoliquiritin apioside *	C_26_H_30_O_13_	[M − H]^−^	549.1647	549.1614	417.1202, 297.0806	6.06	+	+	+
							255.0676, 235.0085				
23	24.225	Ononin *	C_22_H_22_O_9_	[M + H]^+^	431.1334	431.1337	269.0802	−0.6	+	+	+
24	25.225	Ginsenoside Re *	C_48_H_82_O_18_	[M + COOH]^−^	991.5519	991.5483	945.5455, 783.4920, 637.4347, 475.3812	3.78	+	+	+
25	26.034	Saussureamine C [3]	C_19_H_26_N_2_O_5_	[M + H]^+^	363.1921	363.1914	300.1598, 258.1450, 185.1288, 159.1172	1.8	+	—	—
26	26.634	Medicarpin-3-O-glucoside *	C_23_H_26_O_10_	[M + NH4]^+^	480.1873	480.1864	301.1065, 267.0698	1.9	+	+	—
27	26.783	Palmatine [1]	C_21_H_22_NO_4_	[M]^+^	352.1559	352.1548	337.1302, 322.1090,	1.32	+	—	—
							308.1255, 294.1106				
28	27.475	Berberine *	C_20_H_18_NO_4_	[M]^+^	336.123	336.1236	321.0974, 292.0959	−0.1	+	+	+
							259.0444, 191.0731				
29	27.675	Isomucronulatol 7-O-glucoside *	C_23_H_28_O_10_	[M − H]^−^	463.1662	463.161	301.1107, 286.0867, 135.0453, 121.0293	11.27	+	+	—
30	28.174	Calycosin *	C_16_H_12_O_5_	[M + H]^+^	285.0764	285.0757	137.0227	2.29	+	+	+
31	28.982	Saussureamine B [3]	C_20_H_27_NO_4_	[M + H]^+^	346.2038	346.2013	300.1955, 228.0705	7.29	+	+	+
32	29.231	13-Methylberberine [1]	C_21_H_20_NO_4_	[M]^+^	350.1393	350.1392	335.1349, 320.0913, 306.1095, 292.0973	1.76	+	—	—
33	30.283	Saussureamine A [3]	C_20_H_29_NO_4_	[M + H]^+^	348.2172	348.2169	—	0.76	+	—	—
34	31.674	Ginsenoside Rf *	C_42_H_72_O_14_	[M + COOH]^−^	845.4924	845.4904	799.4917, 637.4374,	2.49	+	+	+
							475.3843, 261.0460				
35	31.932	Atractylenolide II [5]	C_15_H_20_O_2_	[M + H]^+^	233.1535	233.1536	215.1424, 287.1474	−0.46	+	+	+
36	31.974	Genistein *	C_15_H_10_O_5_	[M + H]^+^	271.0608	271.0601	153.0176	2.59	+	+	+
37	32.973	Kaempferol *	C_15_H_10_O_6_	[M − H]^−^	285.0428	285.0405	300.9086, 206.9188, 112.9845, 68.9945	8.17	+	—	—
38	33.673	Ginsenoside Rb1 *	C_54_H_92_O_23_	[M − H]^−^	1107.5948	1107.5957	945.5422, 783.4917,	0.78	+	+	+
							621.4383, 323.0992				
39	34.572	Ginsenoside Rc	C_53_H_90_O_22_	[M + COOH]^−^	1123.5902	1123.5906	1077.5841, 945.5428	−0.35	+	+	—
							783.4920, 621.4423				
40	35.222	Ginsenoside Ro *	C_48_H_76_O_19_	[M − H]^−^	955.4978	955.4908	793.4398, 731.4391	7.31	+	+	+
							613.3755, 523.3787				
41	35.673	Ginsenoside Rb2 *	C_53_H_90_O_22_	[M + COOH]^−^	1123.5943	1123.5906	1077.5832, 945.5418, 783.4930, 621.4456	3.45	+	+	—
42	35.974	Astragaloside IV *	C_41_H_68_O_14_	[M + COOH]^−^	829.4605	829.4591	783.4255, 621.4033, 489.3596, 161.0457	1.77	+	+	+
43	36.073	Ginsenoside Rb3 *	C_53_H_90_O_22_	[M + COOH]^−^	1123.5921	1123.5906	1077.5823, 945.5408	1.41	+	+	—
							783.4953, 621.4437				
44	36.082	Isosinensetin [4]	C_20_H_20_O_7_	[M + H]^+^	373.1284	373.1282	358.1035, 343.0801	0.59	+	+	—
							315.0875				
45	36.424	Astragaloside III *	C_41_H_68_O_14_	[M + COOH]^−^	829.4611	829.4591	783.4386, 621.8069	2.54	+	—	—
							489.3555, 161.0470				
46	36.623	Formononetin *	C_16_H_12_O_4_	[M − H]^−^	267.0688	267.0663	252.0445, 223.0417	9.39	+	+	+
							195.0456, 135.0048				
47	38.122	Ginsenoside Rd *	C_48_H_82_O_18_	[M + COOH]^−^	991.5486	991.5483	945.5406, 800.4763	0.3	+	+	+
							162.0489, 89.0258				
48	39.372	Glycyrrhizic acid *	C_42_H_62_O_16_	[M − H]^−^	821.4012	821.3965	351.0580, 289.0580, 193.0354, 113.0241	5.7	+	+	+
49	39.479	Sinensetin [4]	C_20_H_20_O_7_	[M + H]^+^	373.1289	373.1282	358.1035, 343.0801	1.94	+	—	—
							315.0875				
50	40.328	Astragaloside II *	C_43_H_70_O_15_	[M + COOH]^−^	871.4603	871.4697	825.4654, 765.4503, 603.3838, 161.0448	−11.34	+	+	—
51	41.722	Saikosaponin A *	C_42_H_68_O_13_	[M + COOH]^−^	825.4681	825.4642	779.4604, 617.4094	5	+	+	+
							303.2334,				
52	43.969	Nobiletin *	C_21_H_22_O_8_	[M + H]^+^	403.1382	403.1387	388.1148, 373.0916, 355.0806, 211.0234	−1.35	+	+	+
53	44.47	Saikosaponin b1/b2	C_42_H_68_O_13_	[M + COOH]^−^	825.4659	825.4642	779.4614, 617.4096	2.18	+	—	—
							471.3505,				
54	45.32	Saikosaponin b1/b2	C_42_H_68_O_13_	[M + COOH]^−^	825.4648	825.4642	779.4623, 617.4112	0.78	+	—	—
							423.3308				
55	46.579	Atractylenolide I [5]	C_15_H_18_O_2_	[M + H]+	231.1384	231.138	213.1264, 185.1300, 163.0744, 157.0999	1.93	+	+	+
56	49.826	Tangeretin *	C_20_H_20_O_7_	[M + H]^+^	373.1298	373.1282	358.1075, 343.0880	4.35	+	+	+
							325.0707, 297.0757				
57	50.169	Astragaloside I *	C_45_H_72_O_16_	[M + H]^+^	869.4888	869.4893	437.3408, 401.3210	−0.59	+	—	—
							217.0699, 157.0489				
58	52.719	20(S)-Ginsenoside Rg3 *	C_42_H_72_O_13_	[M + COOH]^−^	829.4983	829.4955	783.4960, 621.4427	3.58	+	+	—
							459.3891, 161.0461				
59	53.518	20(R)-Ginsenoside Rg3 *	C_42_H_72_O_13_	[M + COOH]^−^	829.4981	829.4955	783.4905, 621.4330	3.32	+	+	+
							459.3858, 161.0450				
60	59.614	Costunolide *	C_15_H_20_O_2_	[M + H]^+^	233.1526	233.1536	215.1419, 187.1475	−4.33	+	—	—
							131.0851, 81.0702				
61	61.715	Dehydrocostus lactone *	C_15_H_18_O_2_	[M + H]^+^	231.1384	231.138	213.1267, 195.1161,	1.93	+	—	—
							185.1320, 143.0846				

Compounds were tentatively identified based on high-resolution MS data, isotopic abundance, MS/MS fragment ions, fragmentation patterns, and comparison with the published literature. * Compounds were confirmed by comparison with authentic reference standards. “+” indicates that the constituent was detected, whereas “—” indicates that the constituent was not detected.

## Data Availability

The data reported in this study have been deposited in the OMIX database of the China National Center for Bioinformation/Beijing Institute of Genomics, Chinese Academy of Sciences (https://ngdc.cncb.ac.cn/omix, accessed on 23 September 2026), under accession number OMIX021161.

## Supplementary Materials

The following supporting information can be downloaded at: https://www.mdpi.com/article/10.3390/ph19101617/s1, Figure S1: Total ion chromatograms of the JYF aqueous extract, serum, and brain tissue samples. (A) Total ion chromatogram of the JYF aqueous extract. (B) Total ion chromatograms of rat serum samples collected after oral administration of JYF. (C) Total ion chromatograms of rat brain tissue samples collected after oral administration of JYF. Chromatograms were acquired by HPLC–HRMS in positive and negative ion modes; Figure S2: Network proximity analysis between targets of circulating JYF constituents and the withdrawal-related module. (A) Degree- and set-size-matched permutation distribution of network distances between JYF constituent targets and the W module. (B) Shortest-path subnetwork connecting JYF targets with their nearest W-associated genes. (C) Constituent-level network proximity between individual JYF constituents and the W module; Figure S3: Network proximity analysis between targets of circulating JYF constituents and the anxiety/depression-related module. (A) Degree- and set-size-matched permutation distribution of network distances between JYF constituent targets and the AD module. (B) Shortest-path subnetwork connecting JYF targets with their nearest AD-associated genes. (C) Constituent-level network proximity between individual JYF constituents and the AD module; Figure S4: Network proximity analysis between targets of circulating JYF constituents and the sleep disorder-related module. (A) Degree- and set-size-matched permutation distribution of network distances between JYF constituent targets and the S module. (B) Shortest-path subnetwork connecting JYF targets with their nearest S-associated genes. (C) Constituent-level network proximity between individual JYF constituents and the S module; Figure S5: Network proximity analysis between targets of circulating JYF constituents and the withdrawal–anxiety/depression shared module. (A) Degree- and set-size-matched permutation distribution of network distances between JYF constituent targets and the WAD module. (B) Shortest-path subnetwork connecting JYF targets with their nearest WAD-associated genes. (C) Constituent-level network proximity between individual JYF constituents and the WAD module; Figure S6: Network proximity analysis between targets of circulating JYF constituents and the withdrawal–sleep disorder shared module. (A) Degree- and set-size-matched permutation distribution of network distances between JYF constituent targets and the WS module. (B) Shortest-path subnetwork connecting JYF targets with their nearest WS-associated genes. (C) Constituent-level network proximity between individual JYF constituents and the WS module; Figure S7: Principal component analysis of the widely targeted brain metabolomics data. PCA score plots of the brain metabolomic profiles across the experimental groups. The close clustering of QC samples indicates good analytical stability and reproducibility of the widely targeted metabolomics platform; Figure S8: Widely targeted metabolomic analysis of brain tissue in JYF-treated mice undergoing diazepam withdrawal. (A) OPLS-DA score plots of brain metabolite profiles for the control versus model comparison (left) and the JYF-M versus model comparison (right). (B) Volcano plots of differential metabolites for the control versus model comparison (left) and the JYF-M versus model comparison (right). (C) Hierarchical clustering heatmaps of differential metabolites identified in the two comparisons. (D) KEGG functional classification of differential metabolites. (E) KEGG pathway enrichment analysis of differential metabolites.

## Abbreviations

AD	Anxiety/depression-related module
AMP	Adenosine monophosphate
BZD	Benzodiazepine
CORT	Corticosterone
DA	Dopamine
DPA	Docosapentaenoic acid
EEG	Electroencephalography
EMG	Electromyography
ESI	Electrospray ionization
FC	Fold change
GABA	γ-Aminobutyric acid
Glu	Glutamate
Gln	Glutamine
HRMS	High-resolution mass spectrometry
JYF	*Panax quinquefolius*-containing herbal extract
KEGG	Kyoto Encyclopedia of Genes and Genomes
L-DOPA	3,4-Dihydroxyphenylalanine
mTOR	Mammalian target of rapamycin
NE	Norepinephrine
NREM	Non-rapid eye movement
NREMS	Non-rapid eye movement sleep
OPLS-DA	Orthogonal partial least squares discriminant analysis
OXA	Orexin A
PCA	Principal component analysis
PFA	Paraformaldehyde
PPI	Protein–protein interaction
QC	Quality control
REM	Rapid eye movement
REMS	Rapid eye movement sleep
S	Sleep disorder-related module
SWD	Spike-wave discharge
TCA cycle	Tricarboxylic acid cycle
VIP	Variable importance in projection
W	Withdrawal syndrome-related module
WAD	Withdrawal–anxiety/depression shared module
WADS	Withdrawal–anxiety/depression–sleep disorder common module
WS	Withdrawal–sleep disorder shared module
