# Supplementary material for: Interventional Effects and Neurometabolic Regulation of a Panax quinquefolius-Containing Herbal Extract (JYF) in Diazepam Withdrawal Syndrome in Mice
Source: Pharmaceuticals (Basel). 2026 Oct 12;19(10):1617. doi: 10.3390/ph19101617 (PMC13708265; doi:10.3390/ph19101617)
Supplement: Supplementary file 1 [file pharmaceuticals-19-01617-s001.zip › pharmaceuticals-4593545-supplementary.pdf]

Supplementary Figure Legends

Figure S1. Total ion chromatograms of the JYF aqueous extract and serum and brain tissue samples collected from rats after oral administration of JYF.

(**A**) Total ion chromatograms of the JYF aqueous extract; (**B**) Total ion chromatograms of rat serum samples collected after oral administration of JYF; (**C**) Total ion chromatograms of rat brain tissue samples collected after oral administration of JYF. Chromatograms were acquired by HPLC-HRMS in positive and negative ion modes.

Figure S2. Network proximity analysis between targets of circulating JYF constituents and the withdrawal-related module.

(**A**) Degree-and set-size-matched permutation distribution of network distances between JYF constituent targets and the W module; (**B**) Shortest-path subnetwork connecting JYF targets with their nearest W-associated genes; (**C**) Constituent-level network proximity between individual JYF constituents and the W module.

Figure S3. Network proximity analysis between targets of circulating JYF constituents and the anxiety/depression-related module.

(**A**) Degree-and set-size-matched permutation distribution of network distances between JYF constituent targets and the AD module; (**B**) Shortest-path subnetwork connecting JYF targets with their nearest AD-associated genes; (**C**) Constituent-level network proximity between individual JYF constituents and the AD module.

Figure S4. Network proximity analysis between targets of circulating JYF constituents and the sleep disorder-related module.

(**A**) Degree- and set-size-matched permutation distribution of network distances between JYF constituent targets and the S module. (**B**) Shortest-path subnetwork connecting JYF targets with their nearest S-associated genes. (**C**) Constituent-level network proximity between individual JYF constituents and the S module.

Figure S5. Network proximity analysis between targets of circulating JYF constituents and the withdrawal–anxiety/depression shared module.

(**A**) Degree- and set-size-matched permutation distribution of network distances between JYF constituent targets and the WAD module. (**B**) Shortest-path subnetwork connecting JYF targets with their nearest WAD-associated genes. (**C**) Constituent-level network proximity between individual JYF constituents and the WAD module.

Figure S6. Network proximity analysis between targets of circulating JYF constituents and the withdrawal–sleep disorder shared module.

(**A**) Degree- and set-size-matched permutation distribution of network distances between JYF constituent targets and the WS module. (**B**) Shortest-path subnetwork connecting JYF targets with their nearest WS-associated genes. (**C**) Constituent-level network proximity between individual JYF constituents and the WS module.

Figure S7. Principal component analysis of the widely targeted brain metabolomics data.

PCA score plots of the brain metabolomic profiles across the experimental groups. The close clustering of QC samples indicates good analytical stability and reproducibility of the widely targeted metabolomics platform.

Figure S8. Widely targeted metabolomic analysis of brain tissue in JYF-treated mice undergoing diazepam withdrawal.

(**A**) OPLS-DA score plots of brain metabolite profiles for the control versus model comparison and the JYF-M versus model comparison. (**B**) Volcano plots of differential metabolites for the control versus model comparison and the JYF-M versus model comparison. (**C**) Hierarchical clustering heatmaps of differential metabolites identified in the two comparisons. (**D**) KEGG functional classification of differential metabolites. (**E**) KEGG pathway enrichment analysis of differential metabolites.

Figure S1

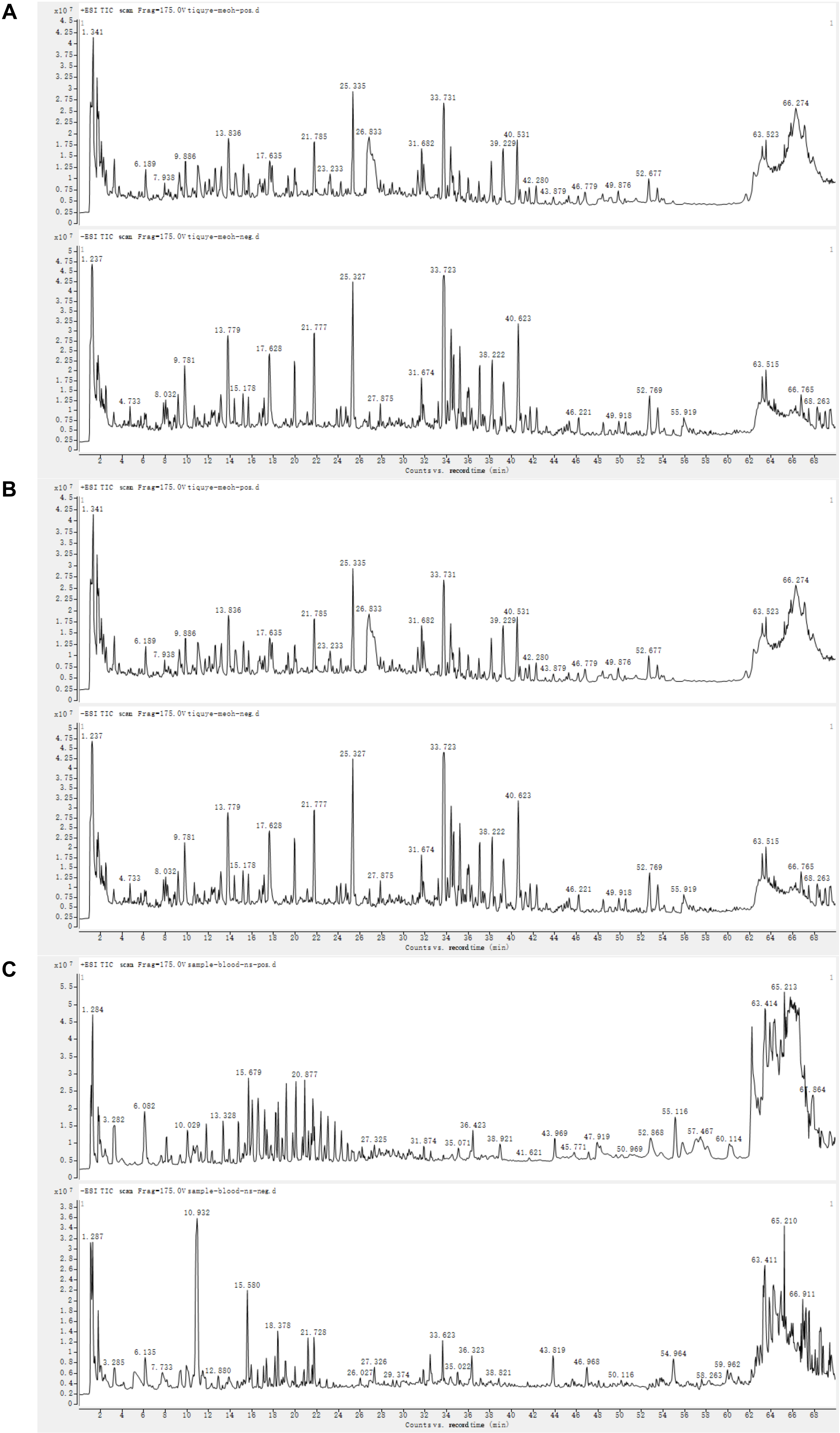
A
x10 7 +ESI TIC scan Frag=175.0V tiquye-meoh-pos.d
1.341
6.189
7.938
9.886
13.836
17.635
21.785
23.233
25.335
26.833
31.682
33.731
39.229
40.531
42.280
43.879
46.779
49.876
52.677
63.523
66.274
x10 7 -ESI TIC scan Frag=175.0V tiquye-meoh-neg.d
1.237
4.733
8.032
9.781
13.779
15.178
17.628
21.777
25.327
27.875
31.674
33.723
38.222
40.623
46.221
49.918
52.769
55.919
63.515
66.765
68.263
Counts vs. record time (min)
B
x10 7 +ESI TIC scan Frag=175.0V tiquye-meoh-pos.d
1.341
6.189
7.938
9.886
13.836
17.635
21.785
23.233
25.335
26.833
31.682
33.731
39.229
40.531
42.280
43.879
46.779
49.876
52.677
63.523
66.274
x10 7 -ESI TIC scan Frag=175.0V tiquye-meoh-neg.d
1.237
4.733
8.032
9.781
13.779
15.178
17.628
21.777
25.327
27.875
31.674
33.723
38.222
40.623
46.221
49.918
52.769
55.919
63.515
66.765
68.263
Counts vs. record time (min)
C
x10 7 +ESI TIC scan Frag=175.0V sample-blood-ns-pos.d
1.284
3.282
6.082
10.029
13.328
15.679
20.877
27.325
31.874
35.071
36.423
38.921
41.621
43.969
45.771
47.919
50.969
52.868
55.116
57.467
60.114
63.414
65.213
x10 7 -ESI TIC scan Frag=175.0V sample-blood-ns-neg.d
1.287
3.285
6.135
7.733
10.932
12.880
15.580
18.378
21.728
26.027
27.326
29.374
33.623
35.022
36.323
38.821
43.819
46.968
50.116
54.964
58.263
59.962
63.411
65.210
66.911
Counts vs. record time (min)

Figure S2. Network proximity analysis between JYF candidate targets and the withdrawal module (W).

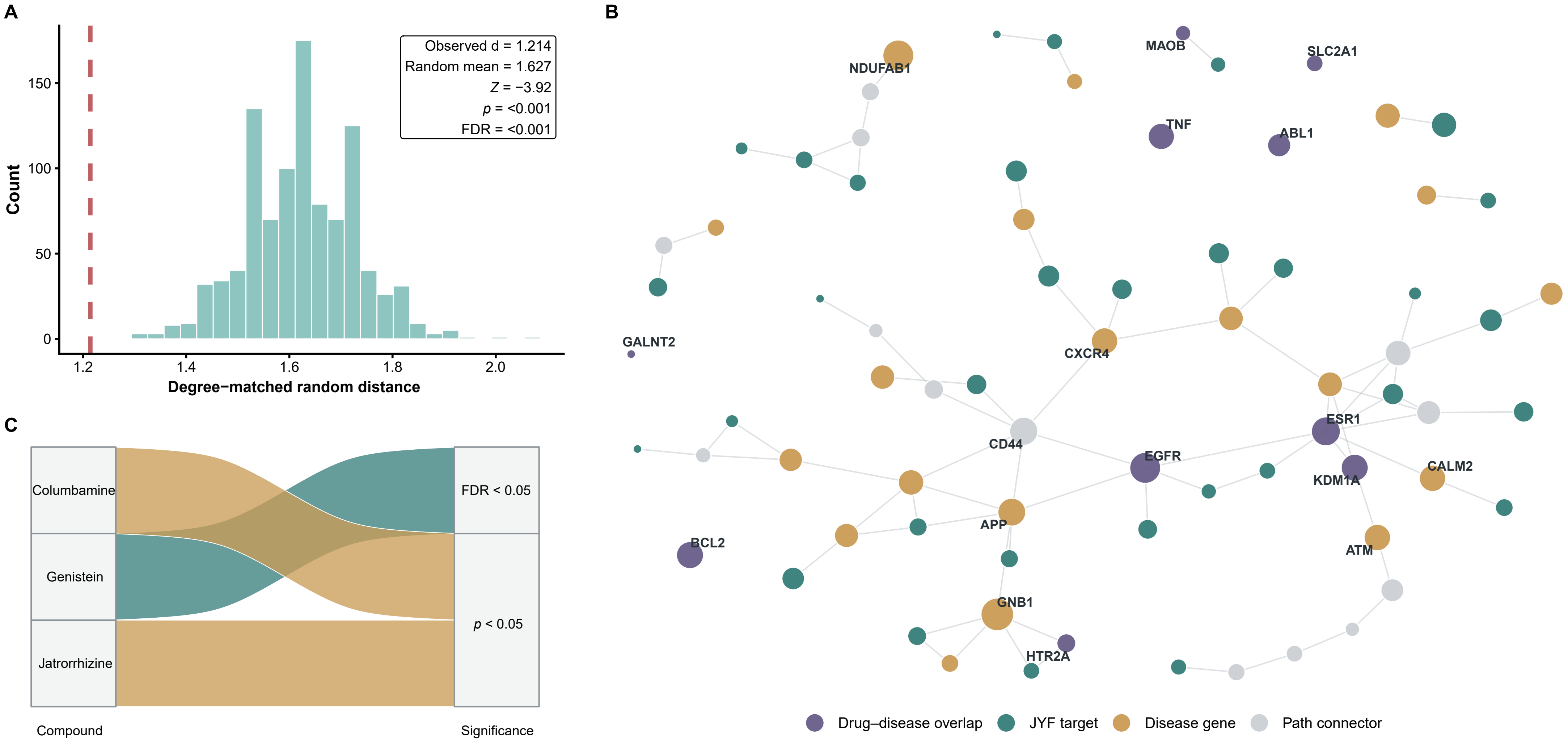

Figure S3. Network proximity analysis between JYF candidate targets and the anxiety/depression module (AD).

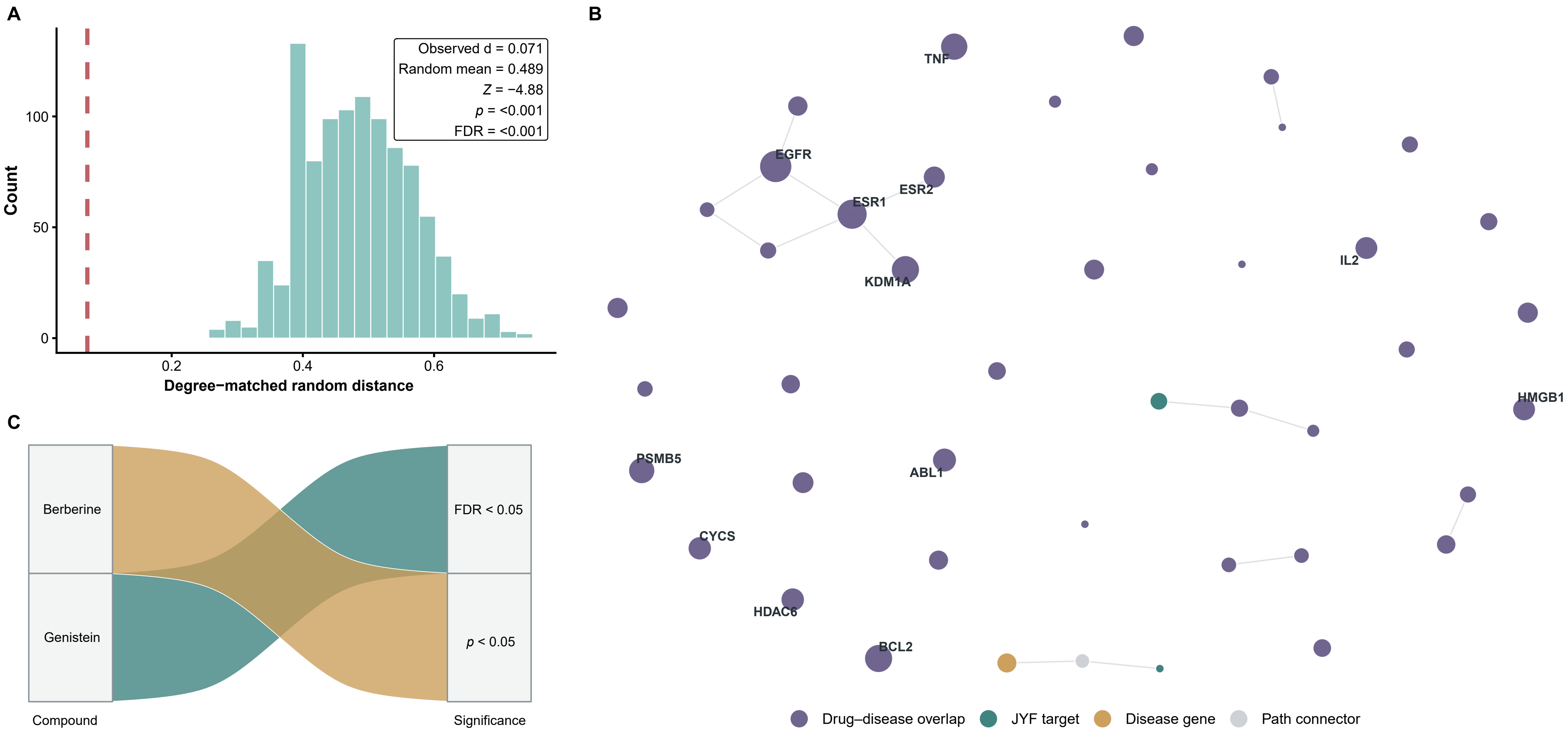

Figure S4. Network proximity analysis between JYF candidate targets and the sleep disturbance module (S).

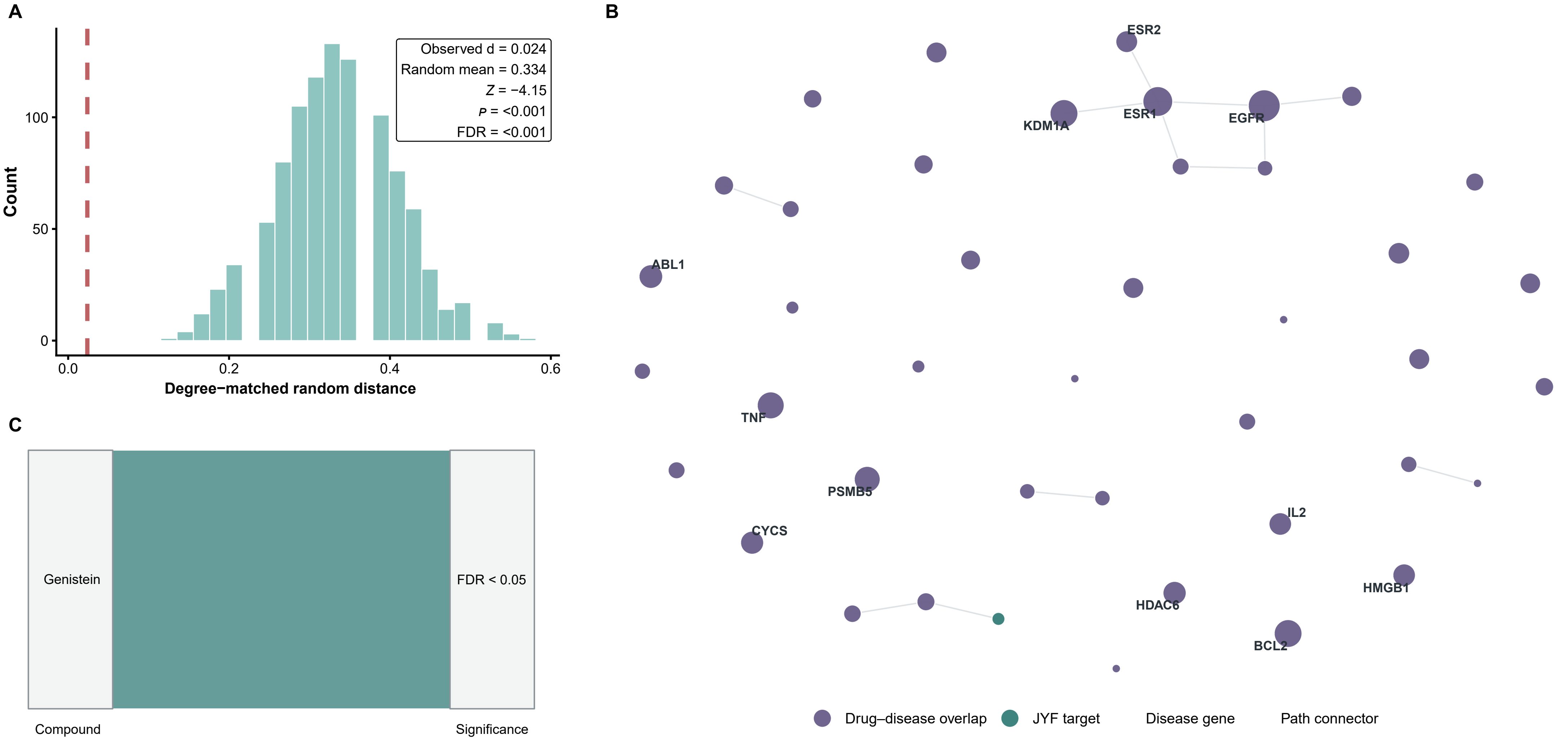

Figure S5. Network proximity analysis between JYF candidate targets and the withdrawal–anxiety/depression shared module (WAD).

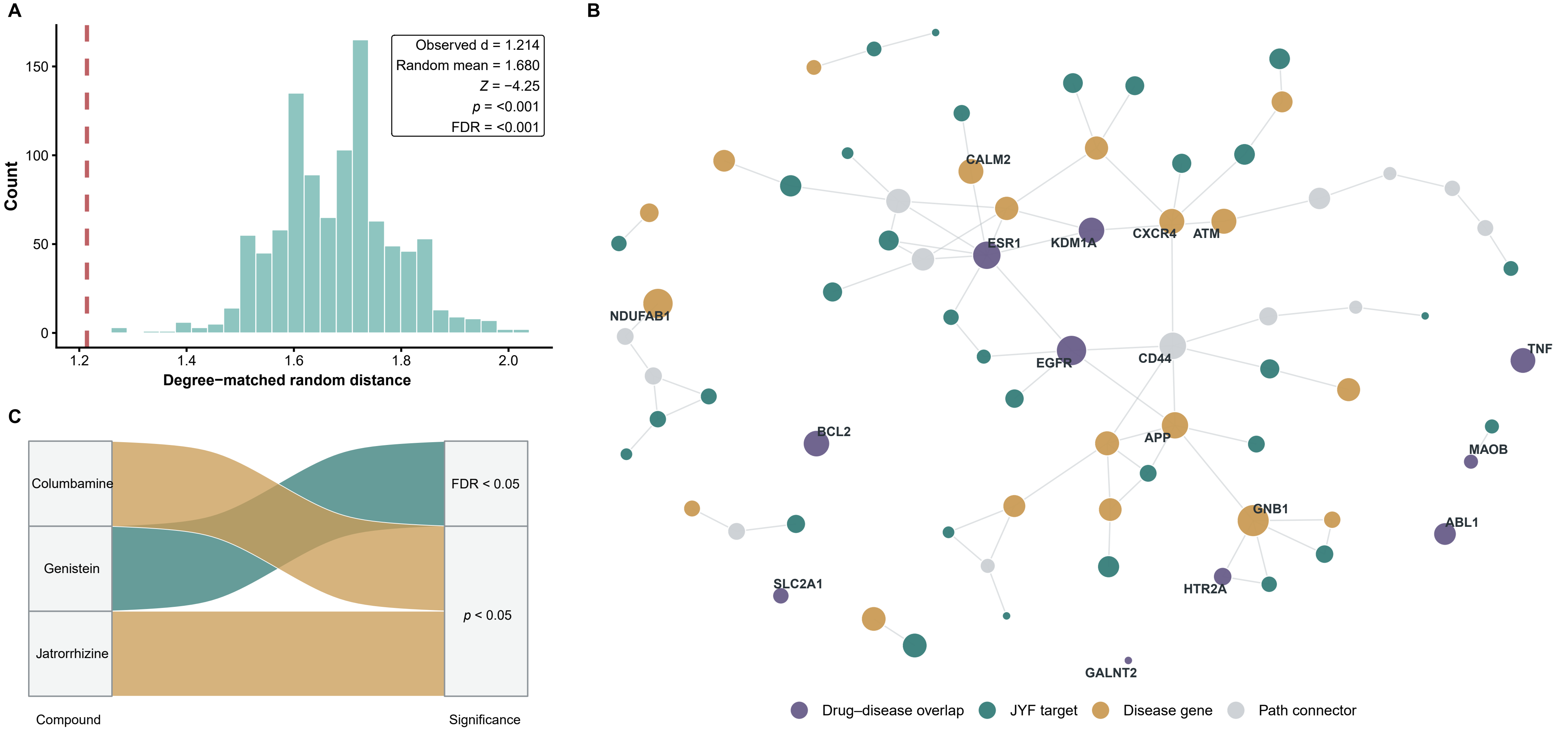

Figure S6. Network proximity analysis between JYF candidate targets and the withdrawal–sleep disturbance shared module (WS).

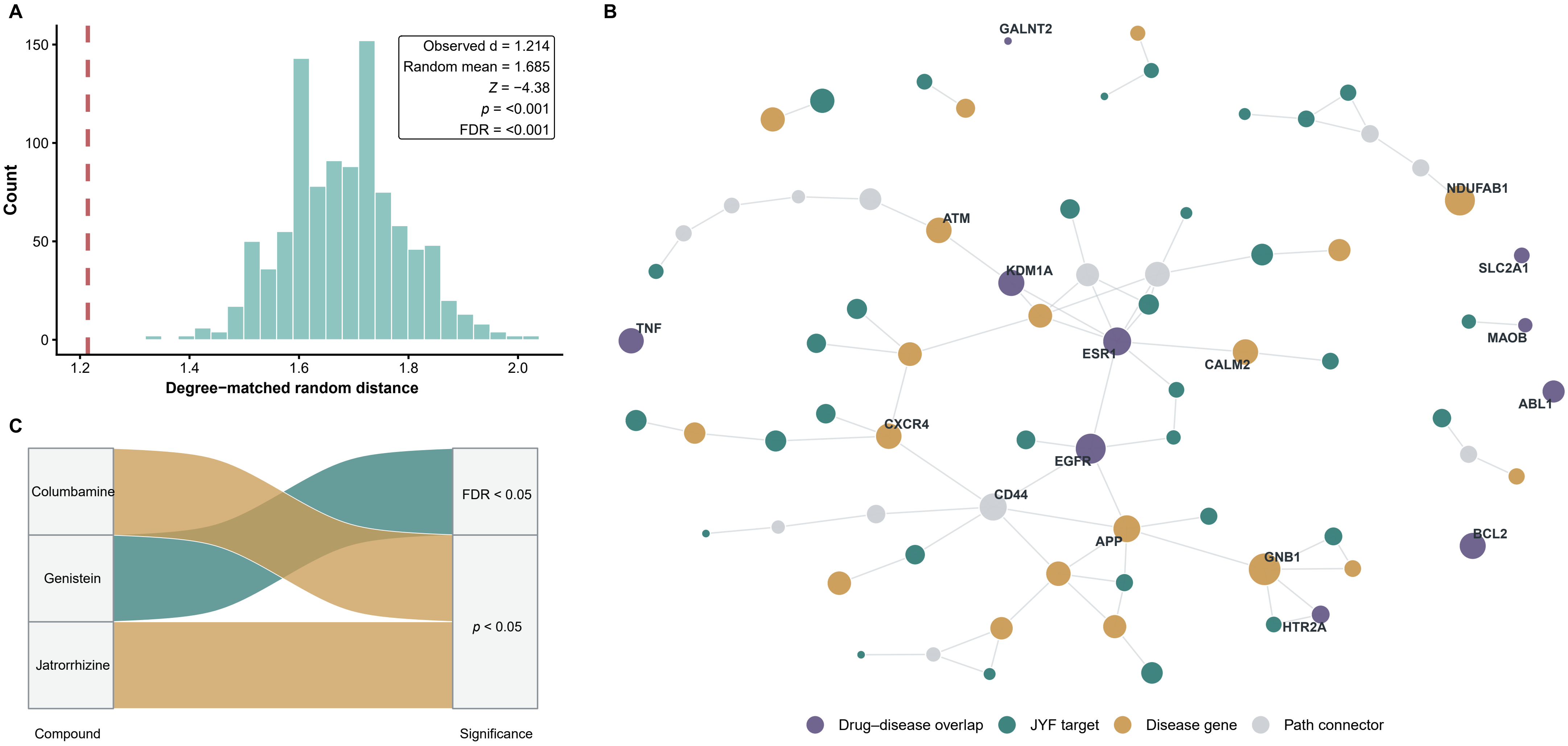

Figure S7

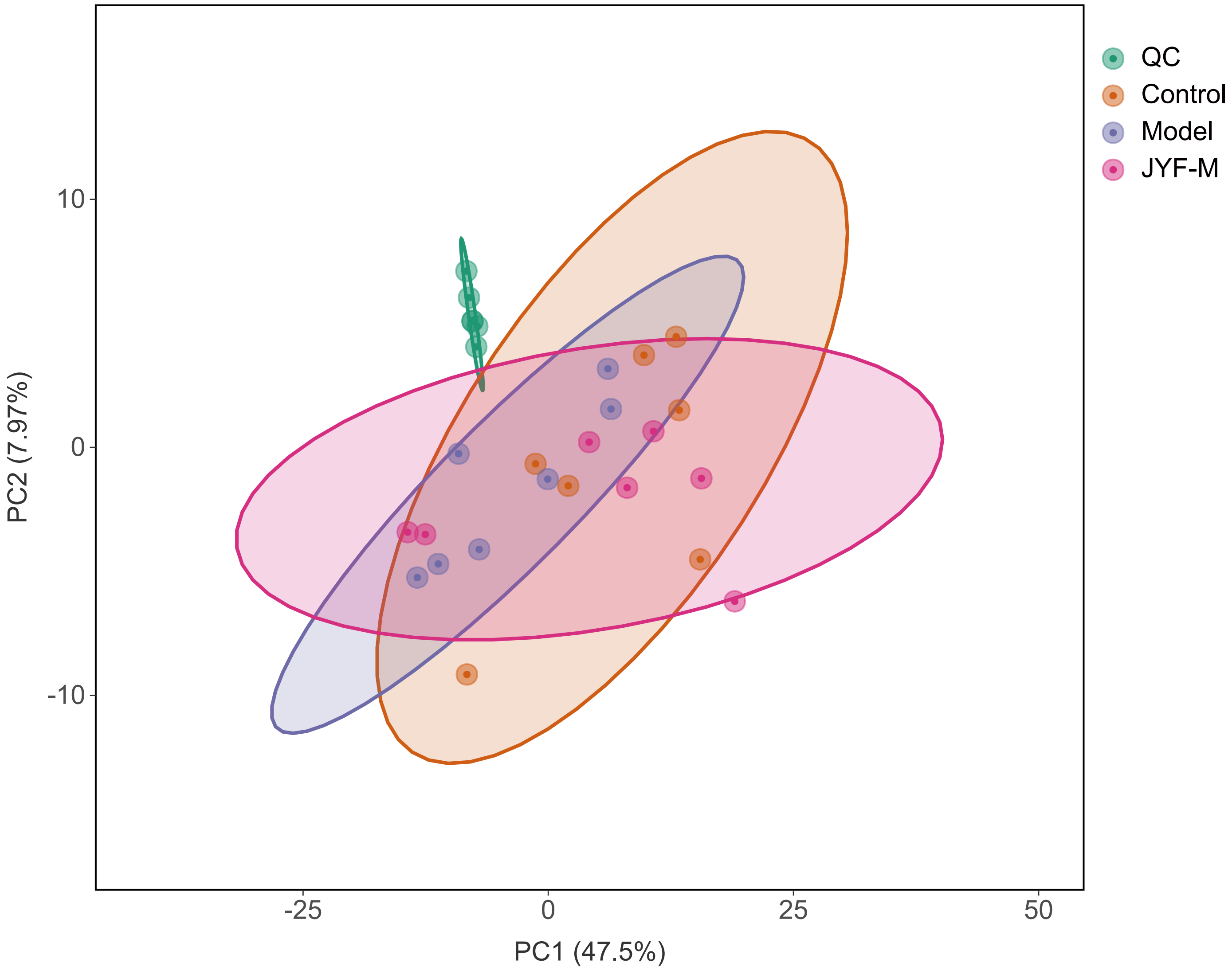

Figure S8

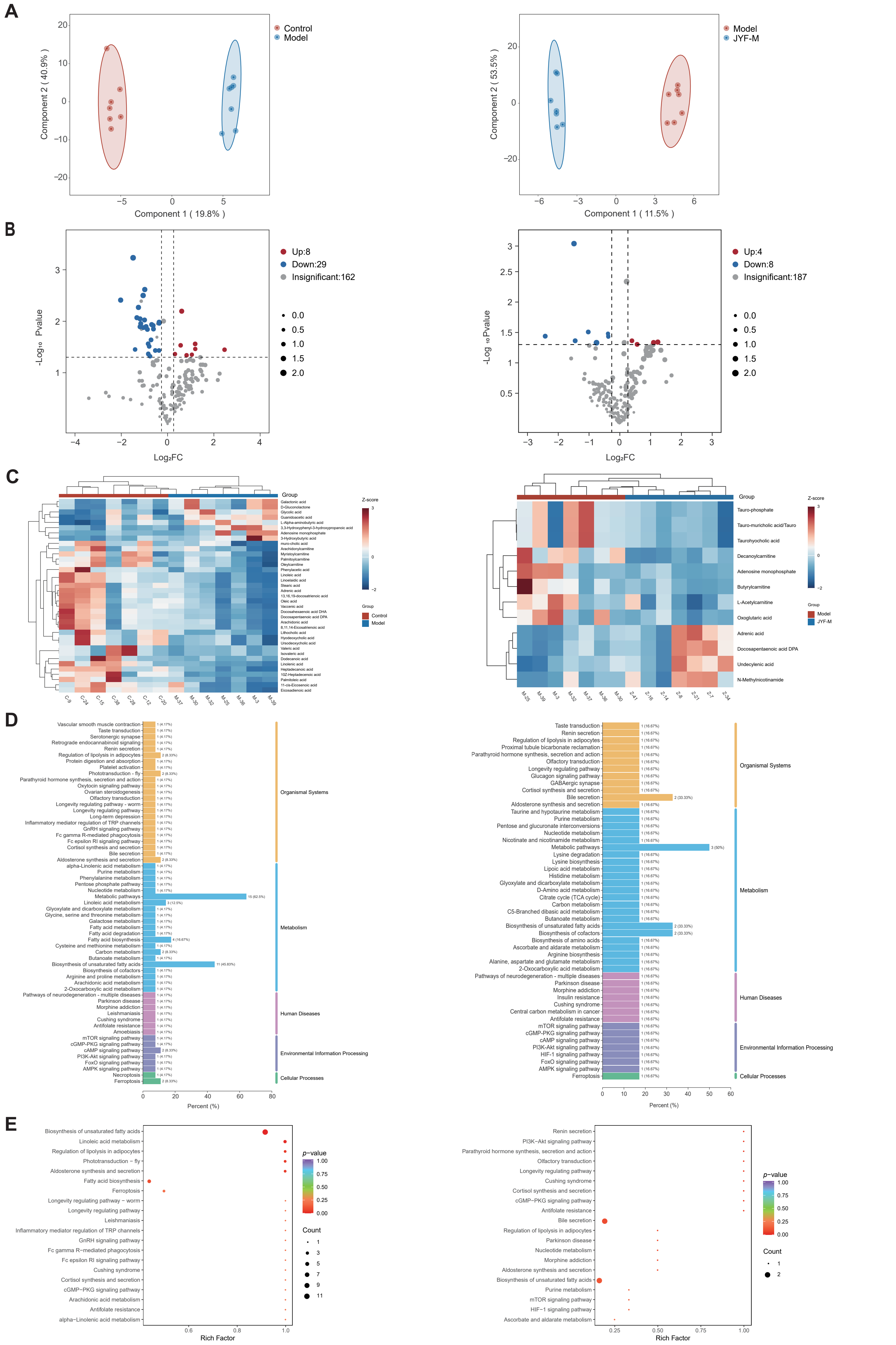
A
Control
Model
Component 1 (19.8%)
Component 2 (40.9%)
Model
JYF-M
Component 1 (11.5%)
Component 2 (53.5%)
B
Up:8
Down:29
Insignificant:162
Log₂FC
-Log₁₀ Pvalue
Up:4
Down:8
Insignificant:187
C
Group
Z-score
Control
Model
JYF-M
D
Organismal Systems
Metabolism
Human Diseases
Environmental Information Processing
Cellular Processes
Percent (%)
E
p-value
Count
Rich Factor
